# Systematics of *Pholidobolus* lizards (Squamata, Gymnophthalmidae) from southern Ecuador, with descriptions of four new species

**DOI:** 10.3897/zookeys.954.50667

**Published:** 2020-07-29

**Authors:** Vanessa Parra, Pedro M. Sales Nunes, Omar Torres-Carvajal

**Affiliations:** 1 Museo de Zoología, Escuela de Ciencias Biológicas, Pontificia Universidad Católica del Ecuador, Avenida 12 de Octubre 1076 y Roca, Quito, Ecuador Pontificia Universidad Católica del Ecuador Quito Ecuador; 2 Departamento de Zoologia, Centro de Biociências, Universidade Federal de Pernambuco, Avenida Professor Moraes Rego, s/n. Cidade Universitária CEP 50670-901, Recife, PE, Brazil Universidade Federal de Pernambuco Recife Brazil

**Keywords:** Andes, Cordillera del Cóndor, diversity, phylogeny, taxonomy

## Abstract

Four new species of *Pholidobolus* lizards are described from poorly explored areas in the Andes of southern Ecuador based on morphological and genetic evidence. Among other morphological characters, *Pholidobolus samek***sp. nov.** and *P. condor***sp. nov.** differ from their congeners in having green dorsolateral stripes on head. Males of *P. condor***sp. nov.** differ from those of *P. samek***sp. nov.** in having reddish flanks and venter. *P. dolichoderes***sp. nov.** is distinguished by having a long neck, with more scales between orbit and tympanum, whereas *P. fascinatus***sp. nov.** is distinguished by lacking enlarged medial scales on collar and a conspicuous vertebral stripe. In addition, the phylogenetic position of the new species is inferred using DNA sequences of mitochondrial and nuclear genes. The phylogeny supports strongly monophyly of each of the new species and renders *P. macbrydei* paraphyletic and split into six subclades. Available data suggest that the new species have restricted distribution ranges (< 100 km^2^ each), and it is proposed that their classification be as Data Deficient or Critically Endangered species. The results reveal unexpected levels of diversity within *Pholidobolus* in the Andes of southern Ecuador and highlight the importance of improving scientific collections and conservation efforts in this area.

## Introduction

The uplift of the Andes mountains was one of the most influential geological events for the evolution and diversification of the South American biota during the Cenozoic. For example, it created many habitats and microclimates that became important centers of biodiversity and endemism (Pérez-Escobar et al. 2017). Therefore, the evolution of diverse Andean taxa is a complex research topic that has attracted the attention of many scientists (Castoe et al. 2004, Torres-Carvajal et al. 2015, Betancourt et al. 2018, Moravec et al. 2018, Lehr et al. 2019). With more than 250 species, Gymnophthalmidae is one of the most diverse lizard clades in the Neotropics. The uplift of the Andes had a strong influence on the radiation of gymnophthalmid lizards, resulting in high levels of diversity and endemism along the Tropical Andes (Torres-Carvajal et al. 2016; Moravec et al. 2018).

*Pholidobolus* lizards are among the most prominent gymnophthalmids in the northern Andes. They are small (SVL ≤ 60 mm), terrestrial, oviparous, and restricted to the Andes of Colombia, Ecuador, and northern Peru at elevations between 1800 and 4100 m (Hurtado-Gómez et al. 2018; Torres-Carvajal et al. 2014; Venegas et al. 2016). *Pholidobolus* is currently known to include ten species: *P. affinis*, *P. anomalus*, *P. dicrus*, *P. hillisi*, *P. macbrydei*, *P. montium*, *P. paramuno*, *P. prefrontalis*, *P. ulisesi*, and *P. vertebralis*, of which three were described in recent years. Remarkably, *P. anomalus* is the only species in the genus that occurs in southern Peru (Cusco), but its generic identity remains questionable (Torres-Carvajal and Mafla-Endara 2013).

The study of *Pholidobolus* and other gymnophthalmid taxa has been often hampered by the paucity of specimens in collections. For example, the recent description of *P. paramuno* reveals the importance of increased sampling effort in the Paramo ecosystem in the northern Andes of Colombia. Similarly, recent collections in poorly explored areas of the southern Andes of Ecuador yielded new specimens of *Pholidobolus* lizards, which we were unable to assign to any of the currently recognized species. Based on these specimens, here we combine evidence from morphology and DNA sequences to describe four new species of *Pholidobolus* and infer their phylogenetic affinities.

## Materials and methods

### Genetic data

Total genomic DNA was digested and extracted from liver or muscle tissue using a guanidinium isothiocyanate extraction protocol. Tissue samples were first mixed with Proteinase K and a lysis buffer and digested overnight prior to extraction. DNA samples were quantified using a Nanodrop ND-1000 (NanoDrop Technologies, Inc.), re-suspended and diluted to 25 ng/µl in ddH2O prior to amplification.

Using primers and amplification protocols from the literature (Pellegrino et al. 2001; Torres-Carvajal and Mafla-Endara 2013), we obtained 1,493 aligned nucleotides (nt) encompassing three mitochondrial genes, 12S (339 nt), 16S (533 nt), and ND4 (621 nt) from 16 individuals of the four new species herein described, as well as 21 individuals of *Pholidobolus macbrydei*. In addition, we obtained 411 nucleotides of the Dynein Axonemal Heavy Chain 3 (DNAH3) nuclear gene from 65 individuals of *Anadia rhombifera*, *Macropholidus annectens*, *M. huancabambae*, *M. labiopunctatus*, *M. ruthveni*, *Pholidobolus affinis*, *P. dicrus*, *P. hillisi*, *P. macbrydei*, *P. montium*, *P. prefrontalis*, *P. ulisesi*, *P. vertebralis*, and the four new species. DNAH3 was amplified using the primers DNAH3_f1 (GGTAAAATGATAGAAGAYTACTG) and DNAH3_r6 (CTKGAGTTRGAHACAATKATGCCAT). The amplification protocol consisted of 1 cycle of initial denaturation for 5 min at 95 °C, 40 cycles of denaturation for 35s at 94 °C, annealing for 1 min at 72 °C, and extension for 1 min at 72 °C, as well as a final extension for 10 min at 72 °C (Townsend et al. 2008). Positive PCR products were visualized in agarose electrophoretic gels and treated with ExoSAP-IT to remove unincorporated primers and dNTPs. Cycle sequencing reactions were carried out by Macrogen Inc. GenBank accession numbers of sequences generated in this study are shown in Table 1. After incorporating GenBank sequences, our data matrix for phylogenetic analyses contained 74 taxa and 1904 characters.

**Table 1.** Vouchers, locality data, and GenBank accession numbers of taxa included in this study. Sequences added in this study are in bold.

Taxon	Voucher	Locality	GenBank number				GenSeq Nomenclature
			12S	16S	ND4	DNAH3	
*Anadia rhombifera*	QCAZ 11862	QCAZ 11862; Ecuador: Cotopaxi: San Francisco de Las Pampas	KU902135	KU902216	KU902291	**MN849427**	genseq-4
*Macropholidus annectens*	QCAZ 11120	Ecuador: Loja: 15 km E Loja	KC894341	KC894355	KC894369	**MN849430**	genseq-4
	QCAZ 11121	Ecuador: Loja: 15 km E Loja	KC894342	KC894356	KC894370	**MN849431**	genseq-4
*Macropholidus huancabambae*	CORBIDI 10492	Peru: Piura: Huancabamba: Las Pozas	KC894343	KC894357	KC894371	**MN849428**	genseq-4
	CORBIDI 10493	Peru: Piura: Huancabamba: Las Pozas	KC894344	KC894358	KC894372	–	genseq-4
	CORBIDI 10496	Peru: Piura: Huancabamba: Las Pozas	KC894345	KC894359	KC894373	**MN849429**	genseq-4
*Macropholidus labiopunctatus*	CORBIDI 12932	Peru: Piura: Ayabaca	KP874774	KP874826	KP874936	**MN849432**	genseq-4
*Macropholidus ruthveni*	CORBIDI 4281	Peru: Lambayeque: El Totora	KC894354	C894368	C894382	**MN849433**	genseq-4
*Pholidobolus affinis*	QCAZ 9641	Ecuador: Cotopaxi: San Miguel de Salcedo, Cutuchi River	KC894348	C894362	C894376	**MN849435**	genseq-4
	QCAZ 9900	Ecuador: Chimborazo: Colta	KC894349	KC894363	KC894377	–	genseq-4
*Pholidobolus condor* sp. nov.	QCAZ 16788	Ecuador: Morona-Santiago: el Quimi	**MN724005**	**MN720239**	**MN717135**	**MN849464**	genseq-2
	QCAZ 16789	Ecuador: Morona-Santiago: el Quimi	**MN724006**	**MN720240**	**MN717134**	**MN849465**	genseq-2
	QCAZ 16790	Ecuador: Morona-Santiago: el Quimi	**MN724007**	**MN720241**	**MN717136**	**MN849466**	genseq-2
	QCAZ 15844	Ecuador: Morona-Santiago: el Quimi	**MN723996**	**MN720230**	**MN717125**	**MN849434**	genseq-1
*Pholidobolus dicrus*	QCAZ 5304	Ecuador: Morona-Santiago: Guarumales	KP874776	KP874828	KP874938	**MN849436**	genseq-4
	QCAZ 6936	Ecuador: Tungurahua: Río Blanco	–	KP874829	KP874939	**MN849437**	genseq-4
*Pholidobolus dolichoderes* sp. nov.	QCAZ 16349	Ecuador: Cañar: Oña	**MN724000**	**MN720234**	**MN717129**	**MN849459**	genseq-2
	QCAZ 16350	Ecuador: Cañar: Oña	**MN724001**	**MN720235**	**MN717130**	**MN849460**	genseq-2
	QCAZ 16351	Ecuador: Cañar: Oña	**MN724002**	**MN720236**	**MN717131**	**MN849461**	genseq-2
	QCAZ 16352	Ecuador: Cañar: Oña	**MN724003**	**MN720237**	**MN717132**	**MN849462**	genseq-2
	QCAZ 16353	Ecuador: Cañar: Oña	**MN724004**	**MN720238**	**MN717133**	**MN849463**	genseq-1
*Pholidobolus fascinatus* sp. nov.	QCAZ 15118	Ecuador: El Oro: Chillacocha	**MN724017**	**MN720251**	**MN717146**	**MN849476**	genseq-2
	QCAZ 15120	Ecuador: El Oro: Chillacocha	**MN724018**	**MN720252**	**MN717147**	**MN849477**	genseq-1
	QCAZ 15122	Ecuador: El Oro: Chillacocha	**MN724019**	**MN720253**	–	**MN849478**	genseq-2
	QCAZ 15170	Ecuador: El Oro: Chillacocha	**MN724020**	**MN720254**	**MN717148**	**MN849479**	genseq-2
*Pholidobolus hillisi*	QCAZ 4998	Ecuador: Zamora-Chinchipe: near San Francisco Research Station	KP090167	KP090170	KP090173	**MN849438**	genseq-4
	QCAZ 4999	Ecuador: Zamora-Chinchipe: near San Francisco Research Station	KP090169	KP090172	KP090175	**MN849439**	genseq-4
	QCAZ 5000	Ecuador: Zamora-Chinchipe: near San Francisco Research Station	KP090168	KP090171	KP090174	**MN849440**	genseq-4
“*Pholidobolus macbrydei*”	KU 218406	Ecuador: Azuay: Cuenca	AY507848	AY507867	AY507886	–	genseq-4
	QCAZ 9914	Ecuador: Azuay: Guablid	KC894352	KC894366	KC894380	**MN849441**	genseq-4
	QCAZ 9932	Ecuador: Azuay: 20 km on road Cuenca-El Cajas	KC894353	KC894367	KC894381	**MN849442**	genseq-4
	QCAZ 9947	Ecuadro: Cañar: Cañar	**MN724012**	**MN720246**	**MN717141**	**MN849474**	genseq-4
	QCAZ 10051	Ecuador: Cañar: Río Guallicanga, quebrada Juncal	**MN724014**	**MN720248**	**MN717143**	**MN849472**	genseq-4
	QCAZ 10052	Ecuador: Cañar: Río Guallicanga, quebrada Juncal	**MN724015**	**MN720249**	**MN717144**	**MN849473**	genseq-4
	QCAZ 10050	Ecuador: Cañar: A 1000 m de la Panamericana Juncal	**MN724013**	**MN720247**	**MN717142**	**MN849471**	genseq-4
	QCAZ 15811	Ecuador: Cañar: Mazar	**MN724021**	**MN720255**	**MN717149**	**MN849480**	genseq-4
	QCAZ 15812	Ecuador: Cañar: Mazar	**MN724022**	**MN720256**	**MN717150**	**MN849481**	genseq-4
	QCAZ 15813	Ecuador: Cañar: Mazar	**MN724023**	**MN720257**	**MN717151**	**MN849482**	genseq-4
	QCAZ 15814	Ecuador: Cañar: Mazar	**MN724024**	**MN720258**	**MN717152**	–	genseq-4
	QCAZ 15815	Ecuador: Cañar: Mazar	**MN724025**	**MN520259**	**MN717153**	–	genseq-4
	QCAZ 15816	Ecuador: Cañar: Mazar	**MN724026**	**MN720260**	**MN717154**	**MN849483**	genseq-4
	QCAZ 15817	Ecuador: Cañar: Mazar	**MN724027**	**MN720261**	**MN717155**	**MN849484**	genseq-4
	QCAZ 15818	Ecuador: Cañar: Mazar	**MN724028**	**MN720262**	**MN717156**	**MN849485**	genseq-4
	QCAZ 15819	Ecuador: Cañar: Mazar	**MN724029**	**MN720263**	**MN717157**	**MN849486**	genseq-4
	QCAZ 15820	Ecuador: Cañar: Mazar	**MN724030**	**MN720264**	**MN717158**	**MN849487**	genseq-4
	QCAZ 15823	Ecuador: Cañar: Mazar	**MN724031**	**MN720265**	**MN717159**	**MN849488**	genseq-4
	QCAZ 15824	Ecuador: Cañar: Mazar	**MN724032**	**MN720266**	**MN717160**	**MN849489**	genseq-4
	QCAZ 6945	Ecuador: Loja: Jimbura	**MN724008**	**MN720242**	**MN717137**	**MN849467**	genseq-4
	QCAZ 6946	Ecuador: Loja: Jimbura	**MN724009**	**MN720243**	**MN717138**	**MN849468**	genseq-4
“*Pholidobolus macbrydei*”	QCAZ 10054	Ecuadro: Loja: Colambo Yacuri Forest	**MN724016**	**MN720250**	**MN717145**	**MN849475**	genseq-4
	QCAZ 7894	Ecuador: El Oro: Guanazán	**MN724011**	**MN720245**	**MN717140**	**MN849470**	genseq-4
	QCAZ 7891	Ecuador: El Oro: Guanazán	**MN724010**	**MN720244**	**MN717139**	**MN849469**	genseq-4
*Pholidobolus montium*	QCAZ 4051	Ecuador: Pichincha: Quito	KC894346	KC894360	KC894374	**MN849443**	genseq-4
	QCAZ 9044	Ecuador: Pichincha: Tababela	KC894347	KC894361	KC894375	**MN849444**	genseq-4
*Pholidobolus paramuno*	MHUAR 12451	Colombia: Antoquia	MK215018	MK215032	MK215046	–	genseq-4
	MHUAR 12480	Colombia: Antoquia	MK215019	MK215033	MK215047	–	genseq-4
	MHUAR 12481	Colombia: Antoquia	MK215020	MK215034	MK215048	–	genseq-4
*Pholidobolus prefrontalis*	QCAZ 9908	Ecuador: Chimborazo: Alausí	KC894350	KC894364	KC894378	–	genseq-4
	QCAZ 9951	Ecuador: Chimborazo: Tixán	KC894351	KC894365	KC894379	**MN849448**	genseq-4
*Pholidobolus samek* sp. nov.	QCAZ 14954	Ecuador: Zamora Chinchipe: Cerro Plateado	**MN723997**	**MN720231**	**MN717126**	**MN849445**	genseq-2
	QCAZ 14955	Ecuador: Zamora Chinchipe: Cerro Plateado	**MN723998**	**MN720332**	**MN717127**	**MN849446**	genseq-1
	QCAZ 14956	Ecuador: Zamora Chinchipe: Cerro Plateado	**MN723999**	**MN720233**	**MN717128**	**MN849447**	genseq-2
*Pholidobolus ulisesi*	CORBIDI 12735	Peru: Cajamarca: Jaen: Huamantanga Forest	KP874787	KP874839	KP874948	**MN849449**	genseq-4
	CORBIDI 12737	Peru: Cajamarca: Jaen: Huamantanga Forest	KP874788	KP874840	KP874949	–	genseq-4
	CORBIDI 1679	Perú: Chota: La Granja	KP874786	KP874838	KP874947	**MN849450**	genseq-4
*Pholidobolus vertebralis*	QCAZ 10667	Ecuador: Pichincha: Santa Lucía de Nanegal	KP874784	KP874836	KP874946	**MN849455**	genseq-4
	QCAZ 10750	Ecuador: Pichincha: Santa Lucía de Nanegal	KP874785	KP874837	KP874947	**MN849458**	genseq-4
	QCAZ 5057	Ecuador: Carchi: Chilma Bajo	KP874778	KP874830	KP874940	**MN849451**	genseq-4
	QCAZ 8687	Ecuador: Carchi: Chilma Bajo	KP874779	KP874831	KP874941	**MN849452**	genseq-4
	QCAZ 8688	Ecuador: Carchi: Chilma Bajo	KP874780	KP874832	KP874942	**MN849453**	genseq-4
	QCAZ 8689	Ecuador: Carchi: Chilma Bajo	KP874781	KP874833	KP874943	**MN849454**	genseq-4
	QCAZ 8717	Ecuador: Carchi: next to Chilma Bajo	KP874782	KP874834	KP874944	**MN849456**	genseq-4
	QCAZ 8724	Ecuador: Carchi: next to Chilma Bajo	KP874783	KP874835	KP874945	**MN849457**	genseq-4

### Phylogenetic analyses

Data were assembled and aligned in Geneious v5.4.6. (Kearse et al. 2012) under default settings for MAFFT Multiple Alignment (Katoh and Toh 2010). ND4 and DNAH3 sequences were translated into amino acids for confirmation of alignment. The best-fit nucleotide substitution models and partitioning scheme were chosen simultaneously using PartitionFinder v2.1.1 (Lanfear et al. 2012) under the Bayesian Information Criterion (BIC). Genes were combined into a single dataset with four partitions: (i) 1^st^ codon position of ND4 and 12S [GTR + I + G]; (ii) 2^nd^ codon position of ND4, 1^st^ codon and 2^nd^ codon positions of DNAH3 [HKY + I + G]; (iii) 3^rd^ codon position of ND4 [GTR + G]; (iv) 16S and 3^rd^ codon position of DNAH3 [SYM + I + G]. Both maximum likelihood (ML) and Bayesian inference (BI) methods were used to obtain the optimal tree topology of the combined, partitioned dataset using the programs RAxML v.8.2.12 (Stamatakis 2014) and MrBayes v3.2.6 (Ronquist et al. 2012), respectively. The ML analysis was performed under the GTRGAMMA model for all partitions. Nodal support (BS) was assessed with the rapid bootstrapping algorithm under the MRE-based Boot-stopping criterion (252 replicates). For BI analysis, all parameters were unlinked between partitions (except topology and branch lengths), and rate variation (prset ratepr = variable) was invoked. Four independent runs, each with four MCMC chains, were set for ten million generations, sampling every 10,000 generations. All analyses were performed using the CIPRES platform (Miller et al. 2010). Results were analyzed in Tracer 1.6 (Rambaut and Drummond 2007) to assess convergence and effective sample sizes (ESS) for all parameters, based on which the first 10% of trees were removed from each run. The remaining trees were used to calculate posterior probabilities (PP) for each bipartition in a Maximum Clade Credibility Tree. The phylogenetic trees were visualized and edited using FigTree v1.4.2 (Rambaut 2014). In order to address interspecific genetic differentiation, uncorrected genetic distances were calculated in MEGA 7 (Kumar et al. 2016) after removing ambiguous positions for each sequence pair (pairwise deletion option).

### Specimens and morphological data

We examined 98 specimens of *Pholidobolus macbrydei* (Appendix I) and 41 of the new species described herein (see corresponding type series). All specimens are deposited in the herpetological collection at Museo de Zoología, Pontificia Universidad Católica del Ecuador, Quito (**QCAZ**). The following measurements were taken with a digital caliper (to the nearest 0.1 mm):

**AGD** axilla-groin distance;

**HL** head length;

**HW** head width;

**ShL** shank length;

**SVL** and snout-vent length.

Tail length (**TL**) was measured with a ruler. Sex was determined by dissection or by noting the presence of everted hemipenes. We followed the terminology of Montanucci (1973) and Kizirian (1996) for morphological characters.

Because the new species are similar in morphology to *Pholidobolus macbrydei*, we assessed the degree of differentiation among them with a Principal Components Analysis (PCA) in R (R Core Team 2018). The PCA was based on 16 quantitative morphological characters: (1) number of supraoculars (NSO), (2) number of scales along margin of upper jaw (SUJ), (3) number of scales along margin of lower jaw (SLJ), (4) number of gular and jaw scales (SGJ), (5) number of ventrals (SGV), (6) number of dorsals (DEL), (7) number of temporals (NTS), (8) number of scales around body (SAB), (9) number of scales around tail (SAT), (10) number of supradigital scales of third finger (SF3), (11) number of supradigital scales of fifth finger (SF5), (12) number of supradigital scales of third toe (ST3), (13) number of supradigital scales of fourth toe (ST4), (14) number of supradigital scales of fifth toe (ST5), (15) lower eyelid scales (LES), and (16) collar scales (i.e., posterior transverse row of gulars; SGC) (Peters 1964, Montanucci 1973).

Hemipenes were prepared following the procedures described by Manzani and Abe (1988), as modified by Pesantes (1994) and Zaher (1999). Organs were everted after immersion in a potassium hydroxide solution, the retractor muscles were manually separated, and the everted organs filled with blue-stained petroleum jelly. Hemipenes were then immersed in an alcoholic solution of Alizarin Red for 24 hours in order to stain eventual calcified structures (e.g., spines or spicules), in an adaptation proposed by Nunes et al. (2012) on the procedures described by Uzzell (1973) and Harvey and Embert (2008). The terminology of hemipenial structures follows previous literature (Dowling and Savage 1960; Hurtado-Gómez et al. 2018; Nunes et al. 2012; Sánchez-Pacheco et al. 2017; Savage 1997; Venegas et al. 2016).

### Systematics

The taxonomic conclusions of this study are based on the observation of morphological features and color pattern, as well as inferred phylogenetic relationships. We consider this information as species delimitation criteria following a general lineage or unified species concept (de Queiroz 1998; 2007).

The new species share with all known species of *Pholidobolus* the presence of a ventrolateral fold between fore and hind limbs and the absence of a single transparent palpebral disc (Montanucci 1973).

## Results

### Phylogenetic relationships and genetic distances

**Figure 1.**
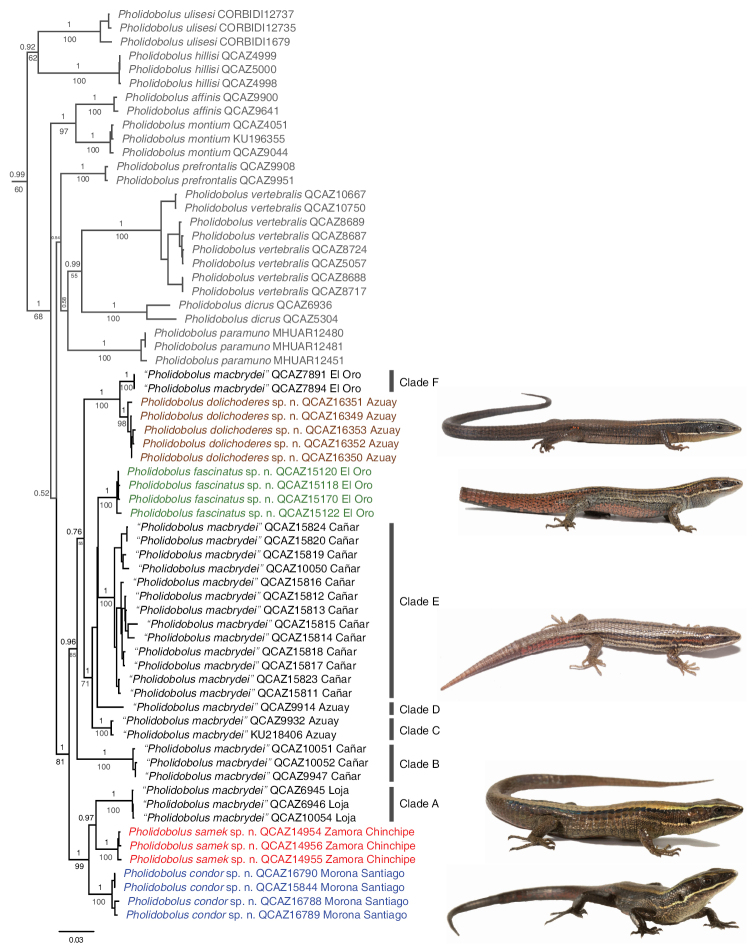
Phylogeny of *Pholidobolus.* Maximum clade credibility tree derived from a partitioned analysis of 1904 bp of mitochondrial and nuclear DNA. Bayesian posterior probabilities are shown above branches and bootstrap values (RAxML analysis) below branches; values ≤ 0.5 and 50, respectively, are not shown. For clarity, outgroup taxa and values on short branches are not shown. Species outside the “*P. macbrydei*” complex are in grey; new species described in this paper are in color matching the distribution records of the map in Figure 7. The species name followed by voucher number and province (“*P. macbrydei*” complex only) are provided for each terminal. Photographs from top to bottom: *P. dolichoderes* sp. nov. holotype, *P. fascinatus* sp. nov. holotype, “*P. macbrydei*” (Clade B) QCAZ 15824, *P. samek* sp. nov. holotype, *P. condor* sp. nov. holotype.

Tree topologies under ML and BI approaches were generally similar; here we describe the maximum clade credibility tree (Fig. 1). Our hypothesis supports the monophyly of *Pholidobolus* (BS= 60, PP = 0.99) and is congruent with previous molecular phylogenies in that *P. ulisesi* and *P. hillisi* form a clade (BS = 62, PP = 0.92) sister to all other congeners (Torres-Carvajal et al. 2015, 2016; Hurtado-Gómez et al. 2018). Following branching order, the strongly supported species pair *P. affinis*, *P. montium* is sister to all remaining species, which form a clade where (*P. prefrontalis* (*P. paramuno* (*P. dicrus*, *P. vertebralis*))) is sister to a subclade containing the new species described in this paper and a paraphyletic *P. macbrydei*. Hereafter we refer to the latter subclade as the “*P. macbrydei*” species complex.

The “*P. macbrydei*” species complex (BS = 81, PP = 1) is divided into two allopatric and strongly supported clades (Fig. 1) that include four new species described below and a paraphyletic “*P. macbrydei*” divided in six subclades (Clades A–F). A southeastern clade (BS = 99, PP = 1) contains *P. condor* sp. nov. as sister to (*P. samek* sp. nov., “*P. macbrydei*” Clade A [Loja province]). The ML tree recovered *P. condor* as sister to “*P. macbrydei*” Clade A with low support (BS = 58). A northwestern clade (BS = 85, PP = 0.96) is composed of “*P. macbrydei*” Clade B from Cañar province as sister to a clade that includes all remaining samples, in which *P. fascinatus* sp. nov. is nested along with “*P. macbrydei*” Clades C, D, and E (Azuay and Cañar provinces) in a strongly supported subclade (BS = 71, PP = 1) sister to the maximally supported (*P. dolichoderes*, “*P. macbrydei*” Clade F [El Oro province]). All new species are strongly supported as monophyletic (BS ≥ 98, PP = 1).

Uncorrected *p*-genetic distances for 16S, 12S, and ND4 are presented in Tables 2, 3, and 4, respectively. Distance values among all recognized species of *Pholidobolus*, the four new species described in this paper, and the six “*P. macbrydei*” clades range between 1 (e.g., *P. condor* sp. nov. vs. *P. samek* sp. nov., Clade C vs. Clade D)–10% (e.g., *P. paramuno* vs. *P. dicrus*) for 12S (average = 5% ± 0.01 SD); 1 (*P. dolichoderes* sp. nov. vs. Clade F)–6% (e.g., *P. dicrus* vs. *P. ulisesi*) for 16S (average = 4% ± 0.01 SD); and 4 (*P. dolichoderes* sp. nov. vs. Clade F)–19% (e.g., *P. dicrus* vs. *P. vertebralis*) for ND4 (average = 14% ± 0.03 SD). Maximum distance values within the “*P. macbrydei*” complex are 5% (Clade A vs. Clade F) for 12S, 4% (*P. condor* sp. nov. vs. Clade F) for 16S, and 14% (Clade A vs. Clade F) for ND4. The genetic distances for the nuclear gene NDH3 are generally low (0–3%, average = 1% ± 0.01 SD).

**Table 2.** Pairwise genetic distances (uncorrected *p*) of 16S DNA sequences among species and clades of *Pholidobolus* included in this study. This analysis involved 66 nucleotide sequences and 533 positions.

	Taxon	1	2	3	4	5	6	7	8	9	10	11	12	13	14	15	16	17
**1**	*Pholidobolus condor* sp. nov.																	
**2**	*Pholidobolus samek* sp. nov.	0.03																
**3**	*Pholidobolus dolichoderes* sp. nov.	0.04	0.03															
**4**	*Pholidobolus fascinatus* sp. nov.	0.03	0.03	0.03														
**5**	Clade A	0.03	0.02	0.04	0.03													
**6**	Clade B	0.03	0.03	0.03	0.03	0.02												
**7**	Clade C	0.02	0.02	0.03	0.02	0.03	0.03											
**8**	Clade D	0.03	0.02	0.03	0.03	0.03	0.03	0.02										
**9**	Clade E	0.03	0.03	0.04	0.02	0.04	0.03	0.02	0.03									
**10**	Clade F	0.04	0.03	0.01	0.04	0.04	0.02	0.03	0.03	0.04								
**11**	*Pholidobolus affinis*	0.04	0.03	0.03	0.03	0.04	0.03	0.03	0.02	0.03	0.03							
**12**	*Pholidobolus dicrus*	0.04	0.04	0.04	0.04	0.04	0.04	0.04	0.03	0.05	0.04	0.05						
**13**	*Pholidobolus hillisi*	0.05	0.05	0.05	0.05	0.04	0.04	0.04	0.03	0.05	0.04	0.04	0.05					
**14**	*Pholidobolus montium*	0.03	0.02	0.03	0.03	0.02	0.02	0.02	0.02	0.04	0.03	0.03	0.04	0.04				
**15**	*Pholidobolus paramuno*	0.04	0.04	0.04	0.03	0.04	0.03	0.03	0.03	0.04	0.04	0.03	0.04	0.04	0.03			
**17**	*Pholidobolus prefrontalis*	0.03	0.03	0.03	0.03	0.03	0.02	0.03	0.03	0.03	0.03	0.03	0.04	0.04	0.03	0.03		
**17**	*Pholidobolus ulisesi*	0.05	0.05	0.05	0.05	0.05	0.05	0.06	0.06	0.05	0.05	0.05	0.06	0.04	0.05	0.05	0.05	
**18**	*Pholidobolus vertebralis*	0.05	0.04	0.04	0.04	0.05	0.04	0.04	0.04	0.05	0.04	0.05	0.05	0.05	0.04	0.04	0.04	0.06

**Table 3.** Pairwise genetic distances (uncorrected *p*) of 12S DNA sequences among species and clades of *Pholidobolus* included in this study. This analysis involved 65 nucleotide sequences and 339 positions.

	Taxon	1	2	3	4	5	6	7	8	9	10	11	12	13	14	15	16	17
**1**	*Pholidobolus condor* sp. nov.																	
**2**	*Pholidobolus samek* sp. nov.	0.01																
**3**	*Pholidobolus dolichoderes* sp. nov.	0.04	0.04															
**4**	*Pholidobolus fascinatus* sp. nov.	0.03	0.03	0.03														
**5**	Clade A	0.03	0.03	0.05	0.04													
**6**	Clade B	0.03	0.03	0.04	0.03	0.03												
**7**	Clade C	0.02	0.02	0.02	0.02	0.03	0.03											
**8**	Clade D	0.02	0.02	0.03	0.02	0.04	0.03	0.01										
**9**	Clade E	0.02	0.02	0.02	0.02	0.03	0.03	0.01	0.01									
**10**	Clade F	0.04	0.04	0.01	0.04	0.05	0.04	0.03	0.03	0.03								
**11**	*Pholidobolus affinis*	0.05	0.04	0.06	0.05	0.05	0.05	0.05	0.05	0.05	0.06							
**12**	*Pholidobolus dicrus*	0.07	0.07	0.08	0.07	0.07	0.07	0.07	0.08	0.07	0.08	0.08						
**13**	*Pholidobolus hillisi*	0.05	0.04	0.05	0.05	0.06	0.05	0.04	0.04	0.05	0.05	0.06	0.08					
**14**	*Pholidobolus montium*	0.03	0.04	0.05	0.05	0.06	0.04	0.04	0.04	0.04	0.05	0.02	0.07	0.05				
**15**	*Pholidobolus paramuno*	0.06	0.06	0.07	0.03	0.07	0.07	0.06	0.06	0.06	0.07	0.07	0.10	0.07	0.06			
**16**	*Pholidobolus prefrontalis*	0.02	0.02	0.04	0.04	0.03	0.03	0.02	0.03	0.03	0.05	0.03	0.05	0.04	0.02	0.06		
**17**	*Pholidobolus ulisesi*	0.04	0.04	0.04	0.04	0.04	0.04	0.03	0.04	0.03	0.04	0.04	0.07	0.04	0.04	0.06	0.03	
**18**	*Pholidobolus vertebralis*	0.08	0.08	0.09	0.08	0.08	0.07	0.08	0.08	0.08	0.09	0.08	0.06	0.10	0.08	0.10	0.06	0.08

**Table 4.** Pairwise genetic distances (uncorrected *p*) of ND4 DNA sequences among species and clades of *Pholidobolus* included in this study. This analysis involved 64 nucleotide sequences and 621 positions.

	Taxon	1	2	3	4	5	6	7	8	9	10	11	12	13	14	15	16	17
**1**	*Pholidobolus condor* sp. nov.																	
**2**	*Pholidobolus samek* sp. nov.	0.08																
**3**	*Pholidobolus dolichoderes* sp. nov.	0.13	0.12															
**4**	*Pholidobolus fascinatus* sp. nov.	0.09	0.10	0.09														
**5**	Clade A	0.09	0.09	0.13	0.11													
**6**	Clade B	0.12	0.12	0.12	0.10	0.13												
**7**	Clade C	0.10	0.11	0.10	0.05	0.12	0.11											
**8**	Clade D	0.11	0.11	0.10	0.05	0.11	0.11	0.06										
**9**	Clade E	0.10	0.10	0.10	0.06	0.11	0.11	0.06	0.06									
**10**	Clade F	0.12	0.12	0.04	0.08	0.14	0.13	0.09	0.10	0.09								
**11**	*Pholidobolus affinis*	0.13	0.14	0.15	0.12	0.13	0.15	0.12	0.11	0.12	0.15							
**12**	*Pholidobolus dicrus*	0.15	0.15	0.15	0.15	0.17	0.15	0.15	0.16	0.16	0.15	0.16						
**13**	*Pholidobolus hillisi*	0.17	0.17	0.17	0.14	0.16	0.18	0.14	0.16	0.14	0.16	0.17	0.19					
**14**	*Pholidobolus montium*	0.13	0.14	0.15	0.12	0.12	0.17	0.13	0.13	0.13	0.14	0.11	0.17	0.17				
**15**	*Pholidobolus paramuno*	0.14	0.14	0.16	0.14	0.15	0.16	0.14	0.13	0.13	0.16	0.14	0.17	0.17	0.15			
**16**	*Pholidobolus prefrontalis*	0.12	0.12	0.14	0.11	0.12	0.14	0.13	0.13	0.12	0.14	0.13	0.16	0.17	0.13	0.13		
**17**	*Pholidobolus ulisesi*	0.15	0.15	0.15	0.16	0.15	0.16	0.14	0.15	0.14	0.15	0.15	0.17	0.16	0.16	0.17	0.16	
**18**	*Pholidobolus vertebralis*	0.17	0.17	0.17	0.17	0.17	0.17	0.15	0.16	0.15	0.17	0.16	0.19	0.18	0.18	0.16	0.17	0.17

### Morphological comparisons among species

**Figure 2.**
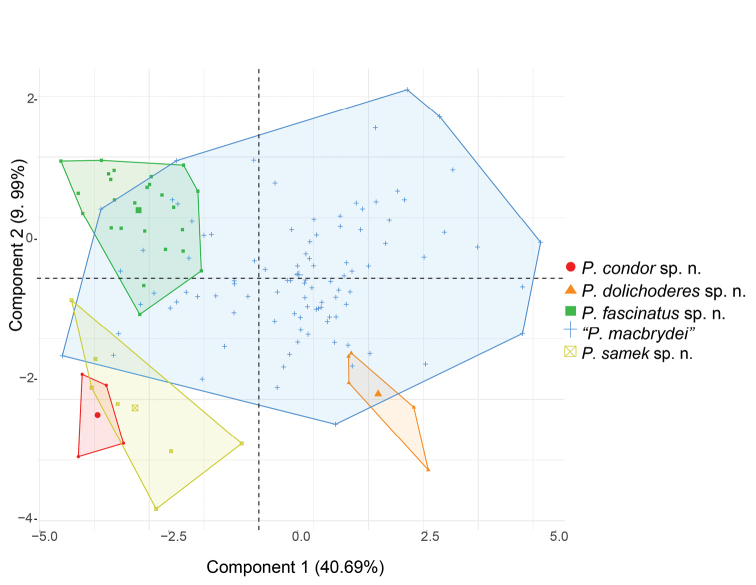
Principal components analysis of 16 morphological variables and 140 specimens of the “*Pholidobolus macbrydei*” species complex. See Table 5 for character loadings on each component.

Two components with eigenvalues > 1.0 were extracted from the PCA (Table 5). These components accounted for 50.7% of the total variation. The highest loadings corresponded to supratympanic temporals (NTS) and number of scales along margin of upper jaw (SUJ) for PC I, and number of scales around the tail (SAT) and number of scales around the body (SAB) for PC II (Table 5). In general, there is wide overlap in morphological space among species of the “*P. macbrydei*” complex (Fig. 2).

**Table 5.** Character loadings, eigenvalues, and percentage of variance explained by Principal Components (PC) I and II. The analysis was based on 16 morphological characters of specimens of “*Pholidobolus macbrydei*”, *Pholidobolus samek* sp. nov., *Pholidobolus condor* sp. nov., *Pholidobolus dolichoderes* sp. nov. and *P. fascinatus* sp. nov. Highest loadings are in bold.

Variable	PCA	
	PC I	PC II
NSO	0.13	-0.11
SUJ	**0.32**	-0.15
SLJ	0.30	-0.10
SGJ	0.29	-0.07
SGV	0.31	0.25
DEL	0.30	0.33
NTS	**0.32**	-0.20
SAB	0.26	**0.39**
SAT	0.18	**0.55**
SF3	0.18	-0.11
SF5	0.22	-0.31
ST3	0.24	0.04
ST4	0.20	-0.12
ST5	0.29	-0.02
LES	-0.05	0.20
SGC	-0.22	0.35
Eigenvalue	6.51	1.60
%	40.69	9.99

### Comparative hemipenial morphology

**Figure 3.**
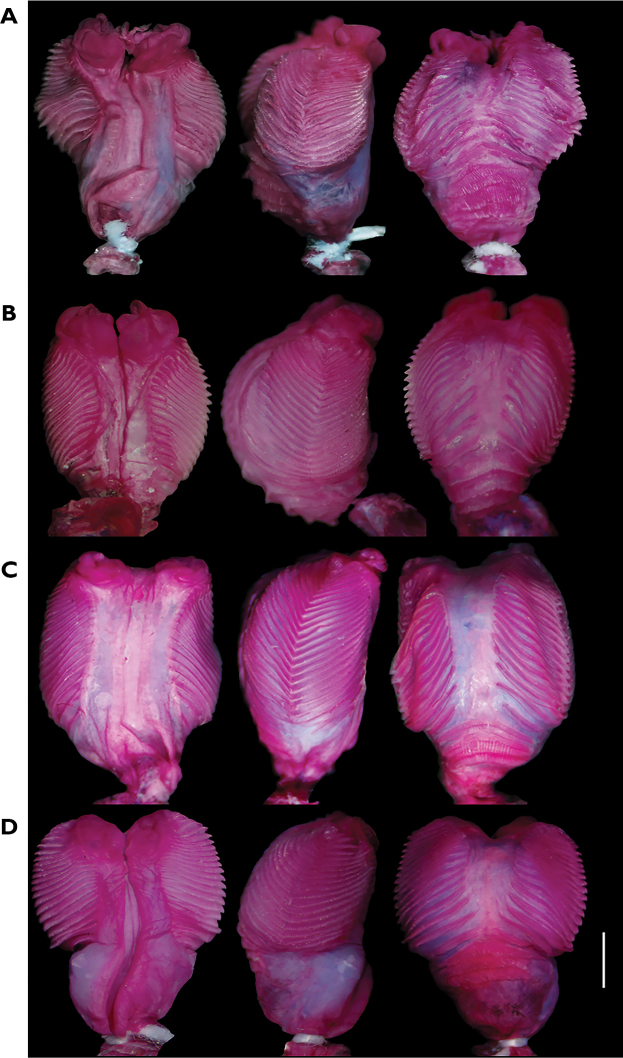
Comparative hemipenial morphology of *Pholidobolus*. Sulcate (left), lateral (center) and asulcate (right) views of: **A***Pholidobolus samek* sp. nov. (QCAZ 14955) **B***Pholidobolus condor* sp. nov. (QCAZ 15844) **C***Pholidobolus dolichoderes* sp. nov. (QCAZ 16353) **D***Pholidobolus fascinatus* sp. nov. (QCAZ 15120). Scale bar: 1 mm.

Hemipenes of holotypes of the four new species described herein are approximately 4–5 mm and 5−7 subcaudal scales long. The organs are fully everted in specimens of *P. fascinatus*, *P. condor*, and *P. samek* and partially everted in *P. dolichoderes*; the hemipenes of the holotype of *P. fascinatus* and *P. condor* are fully expanded, whereas the organs of *P. dolichoderes* and *P. samek* are partially expanded (Fig. 3). All hemipenes have two small lobes detached from the hemipenial body when the organ is fully everted. The hemipenis of *P. condor* presents a distinctive capitular groove originating at the median hemipenial body and extending toward the lobes. The lobes of *P. fascinatus*, *P. condor*, and *P. samek* present folds on their tips, which are not visible in *P. dolichoderes* due to the partial eversion. The hemipenial body is cylindrical in *P. dolichoderes* and *P. condor*, whereas in *P. samek* and *P. fascinatus* the body is conical, with the basis distinctly thinner than the rest of the body. The sulcus spermaticus is broad in *P. fascinatus*, *P. dolichoderes*, and *P. samek*, narrower in *P. condor*; in *P. fascinatus* and *P. condor*, the sulcus spermaticus is deeper than in *P. dolichoderes* and *P. samek*. The sulcus originates medially at the base of the organ and extends in a straight line throughout the body towards the lobes in all species. However, unlike *P. dolichoderes* and *P. condor*, the sulcus originates between thick lips in *P. samek* and *P. fascinatus*. In all species, the sulcus spermaticus bifurcates at the lobular crotch, with each branch extending along the medial face of each lobe.

The sides and borders of the sulcate and asulcate faces are ornamented with a series of roughly equidistant and chevron-shaped flounces, with the chevron vertices aligned medially on each side and directed proximally. All flounces bear calcified comb-like series of spicules, distinctively stained in red with Alizarin. The number of flounces extending along the hemipenial body varies slightly among species: 21 in *P. condor* and *P. samek* and 22 in *P. dolichoderes* and *P. fascinatus*. The base of the asulcate face bears three medial flounces in *P. condor*, *P. dolichoderes*, and *P. samek*, and four in *P. fascinatus*. All species have a conspicuous unevenness forming a bulge along the margins of the asulcate face.

## Systematic accounts

### Pholidobolus samek sp. nov.

Taxon classificationAnimaliaSquamata Gymnophthalmidae

C99EF1F5-646E-5076-B829-E551DE8FE225

http://zoobank.org/431C8AD2-3164-4051-B7DC-1459C4949F51

**Figure 4.**
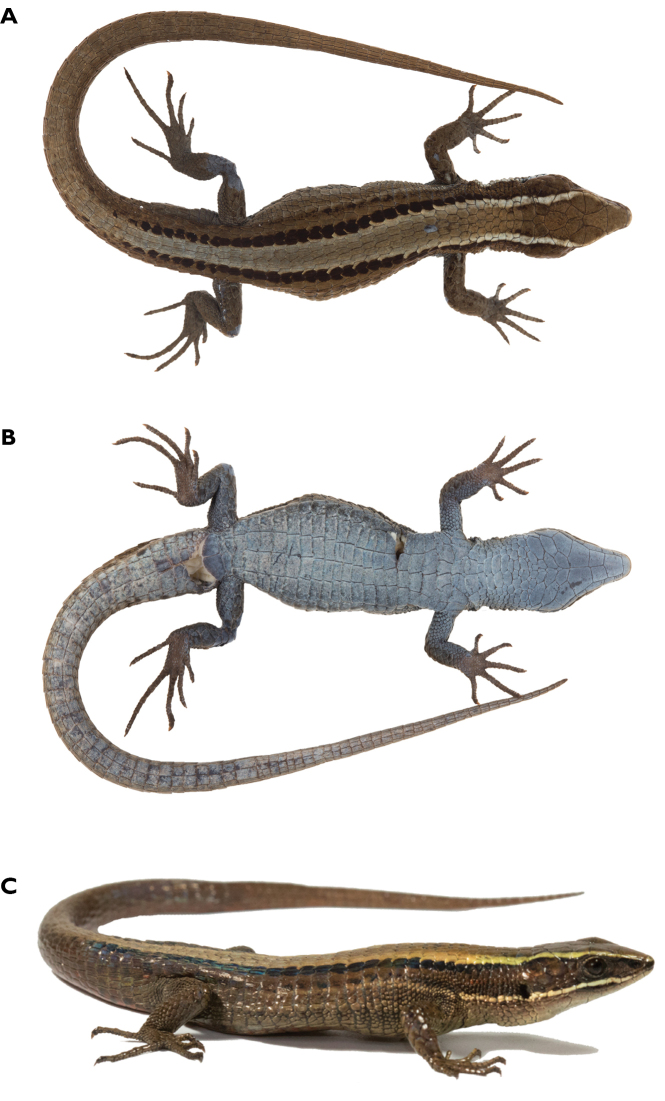
Holotype of *Pholidobolus samek* sp. nov. (QCAZ 14955) in dorsal (**A**), ventral (**B**), and lateral (**C**) views. Male, SVL = 46.7 mm. (A, B): preserved specimen; (C): live specimen. Photographs by Darwin Nuñez and Valeria Chasiluisa.

**Figure 5.**
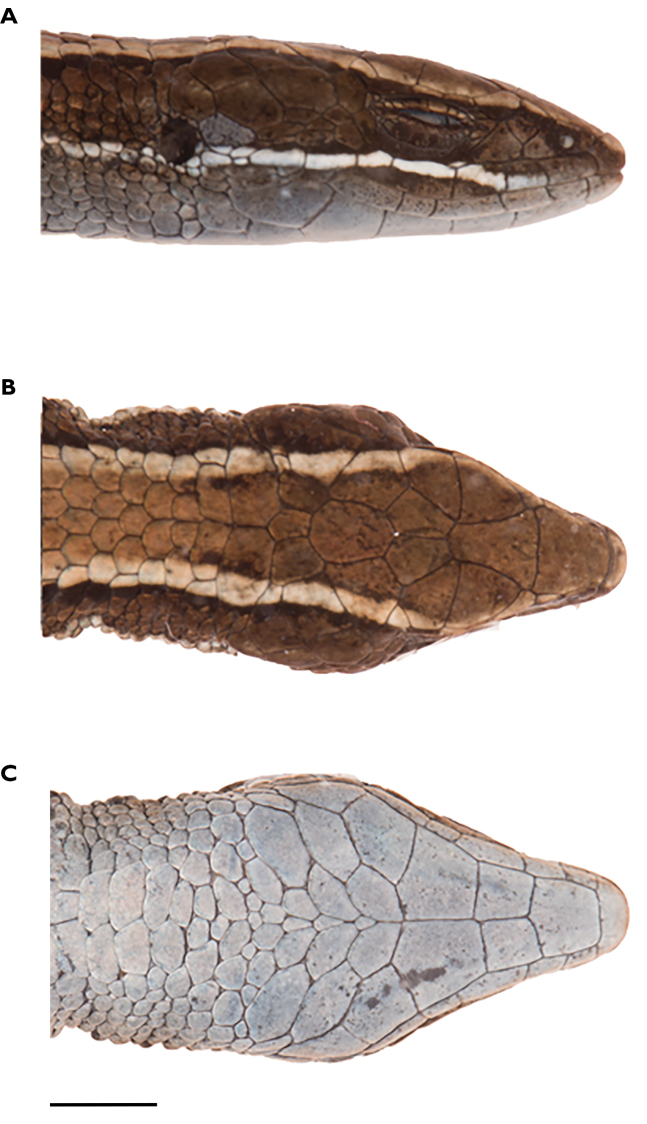
Head of holotype of *Pholidobolus samek* sp. nov. (QCAZ 14955) in lateral (**A**), dorsal (**B**), and ventral (**C**) views. Photographs by Valeria Chasiluisa. Scale bar: 5 mm.

**Figure 6.**
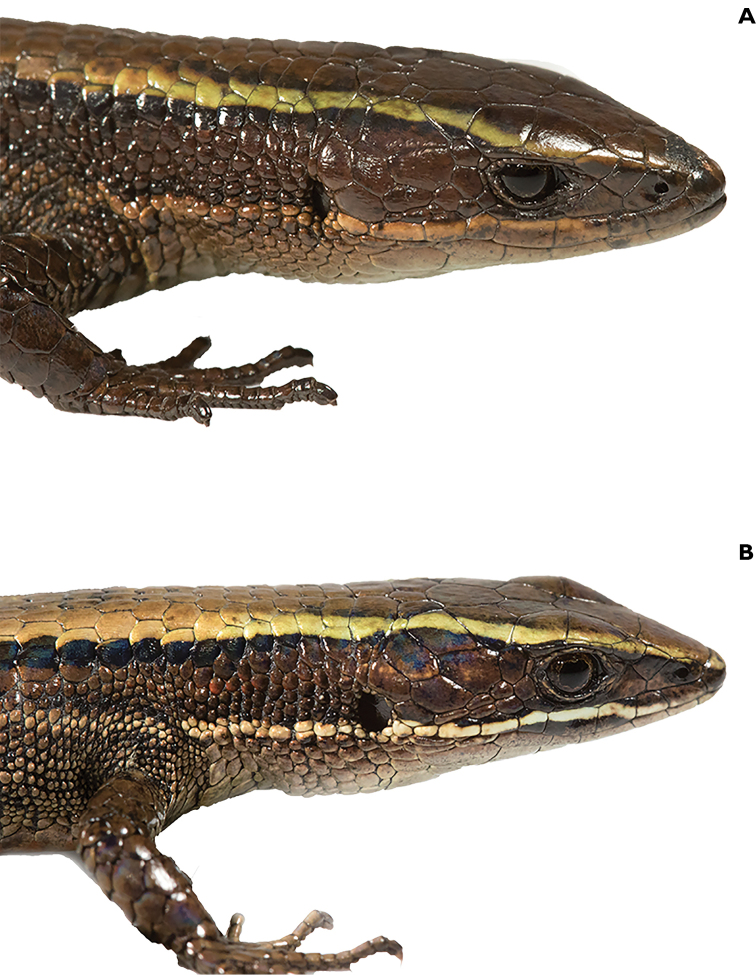
*Pholidobolus samek* sp. nov. in life. **A** Adult female, paratype (QCAZ 14954) **B** adult male, holotype (QCAZ 14955).

Figures 4 , 5 , 6 Proposed standard English name: Green-striped cuilanes Proposed standard Spanish name: Cuilanes de franjas verdes

#### Holotype.

QCAZ 14955 (Figs 4, 5), adult male, Ecuador, Provincia Zamora-Chinchipe, Cerro Plateado Biological Reserve, Cerro Plateado plateau, 4.6159S, 78.7870W, WGS84, 2844 m, 23 September 2016, collected by Diego Almeida, Eloy Nusirquia, Fernando Ayala, Javier Pinto, Alex Achig and Malki Bustos.

#### Paratypes (6).

Ecuador: Provincia Zamora-Chinchipe: QCAZ 14954 (adult female), same data as holotype; QCAZ 14956 (adult female), Cerro Plateado Biological Reserve, 4.6050S, 78.8167W, WGS84, 2320 m, 28 September 2016; QCAZ 14969–70, 14976–77(hatchlings) Cerro Plateado Biological Reserve, 4.6179S, 78.7838W, WGS84, 2873 m, 24 September 2016, same collectors as holotype.

#### Diagnosis.

*Pholidobolus samek* is unique among its congeners, except *P. condor* sp. nov., in having green dorsolateral stripes on the head. However, adult males of *P. samek* differ from those of *P. condor* sp. nov. in having brighter dorsolateral head stripes and lacking a reddish venter. In addition, *P. affinis*, *P. prefrontalis*, *P. macbrydei*, *P. dolichoderes* sp. nov., and *P. montium* differ from *P. samek* (character states of *P. samek* in parentheses) in having a loreal scale frequently in contact with the supralabials (loreal scale not in contact with supralabials), and dorsal scales finely wrinkled (slightly keeled). *Pholidobolus ulisesi* and *P. hillisi* differ from *P. samek* in having a diagonal white bar along the rictal region (white rictal bar absent). *Pholidobolus samek* can be distinguished from *P. dicrus* by lacking a bifurcating vertebral stripe at midbody. *Pholidobolus affinis*, *P. prefrontalis*, *P. dicrus*, *P. hillisi*, and *P. vertebralis* further differ from *P. samek* in having well defined prefrontal scales (if present, prefrontal scales poorly differentiated). Additionally, *P. samek* has fewer dorsal scales (27−29) than *P. affinis* (45−55), *P. montium* (35−50), *P. prefrontalis* (37−46), *P. macbrydei* (31−43), *P. fascinatus* sp. nov. (32−37), and *P. dolichoderes* sp. nov. (35−40). *Pholidobolus samek* can be further distinguished from *P. fascinatus* by having widened medial scales on collar, and from *P. dolichoderes* sp. nov. by having fewer temporals (4–5 and 7–9, respectively), fewer ventrals (19–21 and 25–27), and fewer gulars (15–18 and 22–23).

#### Characterization.

(1) Two (rarely three) supraoculars, anteriormost slightly larger than posterior one; (2) prefrontals present or absent; (3) femoral pores absent in both sexes; (4) four to five opaque lower eyelid scales; (5) scales on dorsal surface of neck striated, becoming slightly keeled from forelimbs to tail; (6) two or three rows of lateral granules at midbody; (7) 27–29 dorsal scales between occipital and posterior margin of hindlimb; (8) lateral body fold present; (9) keeled ventrolateral scales on each side absent; (10) dorsum grayish brown with a distinct golden gray middorsal stripe, slender at midbody, becoming pale gray towards tail; (11) labial stripe white or orange; (12) flanks of body dark brown; (13) conical hemipenial body, with sulcus spermaticus originating between thick lips.

#### Description of holotype.

Adult male (QCAZ 14955) (Figs 4, 5); SVL 46.7 mm; TL 80.9 mm; dorsal and lateral head scales juxtaposed, finely wrinkled; rostral hexagonal, 2.06 times as wide as high; frontonasal irregularly quadrangular, wider than long, laterally in contact with nasal, loreal and first superciliary, slightly bigger than frontal; prefrontal scales absent; frontal longer than wide, in contact with one supraocular on the left side, and two on the right side; frontoparietals pentagonal, longer than wide, slightly wider posteriorly, each in contact laterally with supraocular II; interparietal roughly heptagonal; parietals slightly bigger than interparietal, hexagonal, and positioned anterolaterally to interparietal, each in contact anteriorly with supraocular II (and supraocular III on right side) and dorsalmost postocular; postparietals three, medial scale smaller than laterals; seven supralabials, fourth one longest and below center of eye; six infralabials, fourth one shortest and below center of eye; temporals enlarged, irregularly hexagonal, juxtaposed, smooth; two large supratemporal scales, smooth; nasal slightly divided, irregularly pentagonal, longer than high, in contact with rostral anteriorly, first and second supralabials ventrally, frontonasal dorsally, loreal posterodorsally and frenocular posteroventrally; nostril on ventral aspect of nasal, directed lateroposteriorly; loreal rectangular, wider dorsally; frenocular higher than long, in contact with nasal, separating loreal from supralabials; two supraoculars on left side, three on right side (posteriormost much smaller), with the first one being the largest; four elongate superciliaries, first one enlarged, in contact with loreal; palpebral disc divided into four enlarged, pigmented scales; suboculars three (on the left side the medial subocular is fragmented), elongated and homogeneous in size; two postoculars, the dorsalmost wider than the other; ear opening vertically oval, without denticulate margins; tympanum recessed into a shallow auditory meatus; mental semicircular, wider than long; postmental pentagonal, slightly wider than long, followed posteriorly by three pairs of genials, the anterior two in contact medially and the posterior one separated by postgenials; all genials in contact with infralabials; gulars imbricate, smooth, posteriorly widened in two longitudinal rows; posterior row of gulars (collar) with six scales, the medial two widened.

Nuchal scales similar in size to dorsals, except for the anteriormost that are widened; scales on sides of neck small and granular; dorsal scales hexagonal, elongate, imbricate, arranged in transverse rows; scales on dorsal surface of neck striated, becoming progressively keeled from forelimbs to tail; number of dorsal scales between occipital and posterior margin of hindlimbs 27; dorsal scale rows in a transverse line at midbody 26; one longitudinal row of smooth, enlarged ventrolateral scales on each side; dorsals separated from ventrals by two rows of small scales at level of 13^th^ row of ventrals; lateral body fold between fore and hindlimbs present; ventrals smooth, wider than long, arranged in 20 transverse rows between collar fold and preanals; six ventral scales in a transverse row at midbody; subcaudals smooth; axillary region with granular scales; scales on dorsal surface of forelimb striated, imbricate; scales on ventral surface of forelimb granular; two thick, smooth thenar scales; supradigitals (left/right) 3/3 on finger I, 6/7 on II, 8/8 on III, 9/9 on IV, 6/6 on V; supradigitals 3/4 on toe I, 6/6 on II, 10/9 on III, 11/12 on IV, 7/7 on V; subdigital lamellae of fingers I and II single, paired on III (except the four distalmost), paired at base on IV, on finger V all single; subdigital lamellae 5/5 on finger I, 11/12 on II, 15/16 on III, 17/16 on IV, 9/10 on V; subdigital lamellae on toes I and II single, on toe III, IV and V all paired, except for the three distalmost subdigitals; subdigital lamellae 6/6 on toe I, 11/10 on II, 16/15 on III, 21/21 on IV, 14/14 on V; groin region with small, imbricate scales; scales on dorsal surface of hindlimbs smooth and imbricate; scales on ventral surface of hindlimbs smooth; scales on posterior surface of hindlimbs granular; femoral pores absent; preanal pores absent; cloacal plate paired, bordered by four scales anteriorly, of which the two medialmost are enlarged.

Additional measurements (mm) and proportions of the holotype: HL 11.4; HW 7.4; ShL 7.0; AGD 23.9; TL/SVL 1.5; HL/SVL 0.2; HW/SVL 0.2; ShL/SVL 0.1; AGD/SVL 0.5.

#### Color of holotype in life.

Dorsal background from head to base of tail grayish brown, with a golden light brown vertebral stripe extending from occiput to tail; bright green dorsolateral stripes on head; cream white longitudinal stripe extending from first supralabial to shoulder; sides of neck, flanks and limbs dark brown; reddish brown narrow stripe extending from tympanum to arm insertion; ventrolateral region of body grayish brown; throat cream; chest, belly and base of tail cream orange (Figs 4C, 6B).

#### Color of holotype in preservative.

Dorsal background uniformly grayish brown, with a golden-gray vertebral stripe extending from occiput to tail; vertebral stripe wider anteriorly, becoming slightly slender at most posterior part of body; dorsal and lateral surfaces of head brown (rostral, frontonasal, frontal, frontoparietals, and supraoculars); bluish white longitudinal stripe extending from first supralabial to shoulder and fading on flanks; ventrolateral aspect of neck dark brown with a dorsolateral light brown stripe extending posteriorly along flanks to hindlimbs; forelimbs with scattered ocelli (black with white center); flanks grayish brown with two dorsolateral stripes on each side, the dorsal one dark brown and the most ventral one brown diffuse with dark brown spots; tail brown dorsally; ventral surface of head gray, chest and venter dark gray, ventral surface of tail slightly gray, with scattered dark brown marks.

#### Variations.

Measurements and scale counts of *Pholidobolus samek* are presented in Table 6. Supralabials 8/7 (left/right) and temporals five in specimen QCAZ 14956; small and separated prefrontals on both sides in QCAZ 14954 and one prefrontal on right side in QCAZ 14956; little intrusive scales between parietal and postparietal in QCAZ 14954; frontal hexagonal in QCAZ 14956; roughly decagonal interparietal in QCAZ 14954. Usually two scales on posterior cloacal plate, four in QCAZ 14954 and 14956. Male is larger (SVL 46.7 mm, *N* = 1) than females (maximum SVL 45.4 mm, *N* = 2). Hatchlings (QCAZ 14969, 14970, 14976) with eight or seven (QCAZ 14976) posterior gular (collar) scales. Unlike the male holotype, females have an orange-brown longitudinal stripe extending from third supralabial to shoulder and fading on the flanks (Fig. 6).

#### Distribution and natural history.

**Figure 7.**
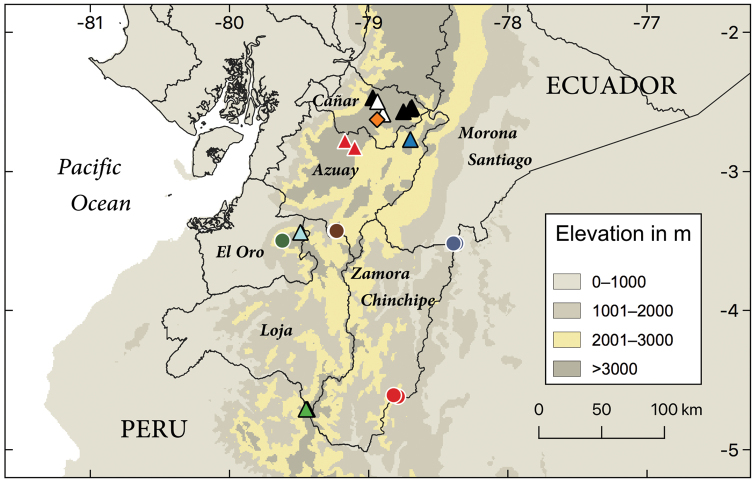
Distribution of samples of the “*Pholidobolus macbrydei*” species complex included in phylogenetic analyses. Circles correspond to four new species described in this paper: *P. samek* sp. nov. (red), *P. condor* sp. nov. (blue), *P. dolichoderes* sp. nov. (brown), and *P. fascinatus* sp. nov. (green). Triangles are “*Pholidobolus macbrydei*” clades as illustrated in the phylogenetic tree (Fig. 1): **A** (green) **B** (white) **C** (red) **D** (blue) **E** (black) **F** (turquoise). Orange diamond corresponds to type locality of *P. macbrydei*. This map was created in QGIS v3.10.

**Figure 8.**
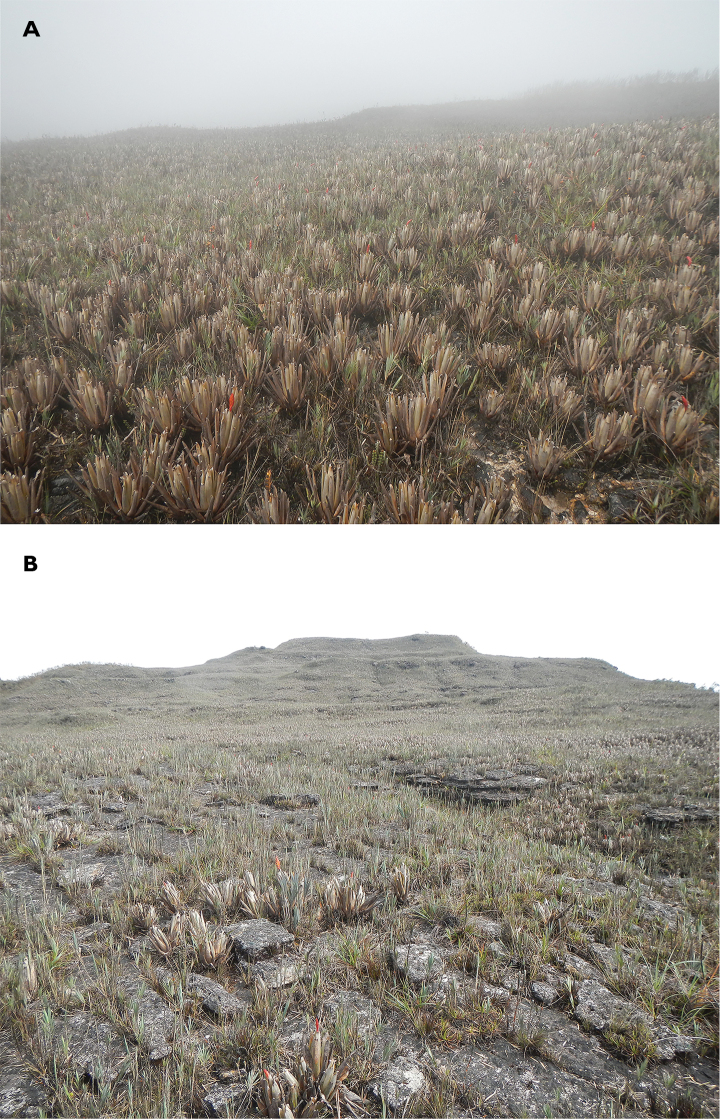
Habitat of *Pholidobolus samek* sp. nov. **A** Vegetation around type locality, Cerro Plateado Biological Reserve, Ecuador **B** habitat where holotype was found. Photographs by Álvaro Pérez.

*Pholidobolus samek* inhabits cloud forests in Cordillera del Cóndor, southeastern Ecuador at elevations between 2324−2844 m (Fig. 7). The new species is known only from Zamora-Chinchipe province, on the sandstone plateaus of Cerro Plateado Biological Reserve. The ground at the type locality is covered with mosses, roots, and bromeliads. Such ground cover is locally known as bamba. All specimens were found active at 11h30–17h00 under stones or terrestrial bromeliads (Fig. 8). Four eggs, collected under flat stones on 24-09-2016, were incubated in sphagnum and perlite in captivity for two months approximately. They were 14.0–14.1 mm long, 8.0–8.5 mm wide, and weighted 0.4 g on average. Hatchlings (QCAZ 14969–70, 14976–77) weighted 0.3 g and were 24.7 mm in SVL on average.

#### Conservation status.

*Pholidobolus samek* is only known from Cordillera del Cóndor. The population size for this species is unknown, but our sampling suggests low abundances. Because of the small known distribution, as well as habitat destruction through mining activities nearby (Van Teijlingen 2016), we suggest assigning *P. samek* to the Critically Endangered category under criteria B1a, b(iii); C1; D, according to IUCN (2012) guidelines.

#### Etymology.

The specific epithet *samek* means green in the Shuar language, in allusion to the green dorsolateral head stripes distinguishing the new species from other congeners. The type locality of *Pholidobolus samek* lies within territory of Shuar indigenous people, who inhabit the Amazonian rainforest in Ecuador and Peru.

#### Remarks.

*Pholidobolus samek* sp. nov. is very similar morphologically and genetically to *P. condor* sp. nov. These species can be easily distinguished from each other by coloration in adult males, although we recognize that our sample size is small (*N* = 7 and 4, respectively) and includes only one adult male per species. However, further evidence supports recognition of *P. samek* and *P. condor* as different species. First, they are reciprocally monophyletic and they are not sister taxa, with *P. samek* being sister to “*P. macbrydei*” Clade A (Fig. 1), which is very different in color patterns from either *P. samek* or *P. condor* (V. Parra and O. Torres-Carvajal, personal observation). Second, unlike the 12S gene (the less variable gene in this study), genetic distances between *P. samek* and *P. condor* for 16S and ND4 are not the lowest (Tables 2 and 4, respectively) within *Pholidobolus*. For example, the 16S distance between *P. samek* and *P. condor* (3%) is the same as the distance between the well-recognized species *P. paramuno* and *P. affinis*. In addition, genetic exchange among *P. samek*, *P. condor* and Clade A is very unlikely as they are isolated from each other on mountaintops above 2000 m (Fig. 7).

### Pholidobolus condor sp. nov.

Taxon classificationAnimaliaSquamata Gymnophthalmidae

B67910B2-60EE-54F5-B489-77705A7EA8A0

http://zoobank.org/BB38EC4E-634D-4728-BA30-412913E7D0E0

**Figure 9.**
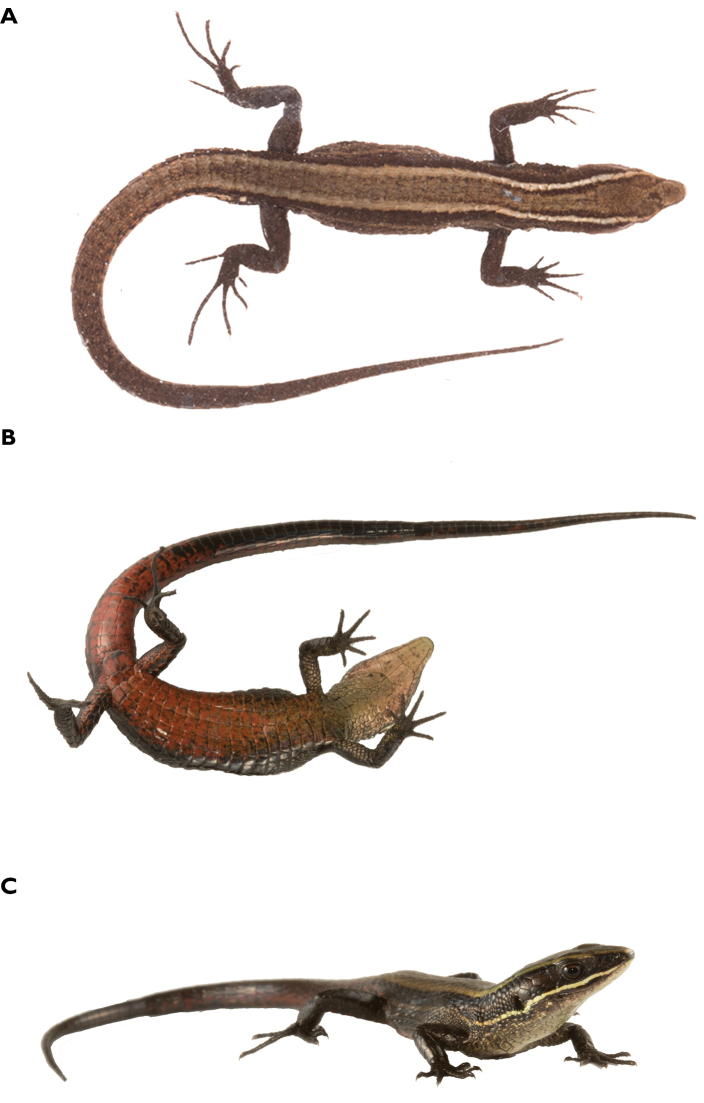
Holotype of *Pholidobolus condor* sp. nov. (QCAZ 15844) in dorsal (**A**), ventral (**B**), and lateral (**C**) views. Male, SVL = 42.7 mm. Preserved specimen (**A**); live specimen (**B, C**). Photographs by Malki Bustos.

**Figure 10.**
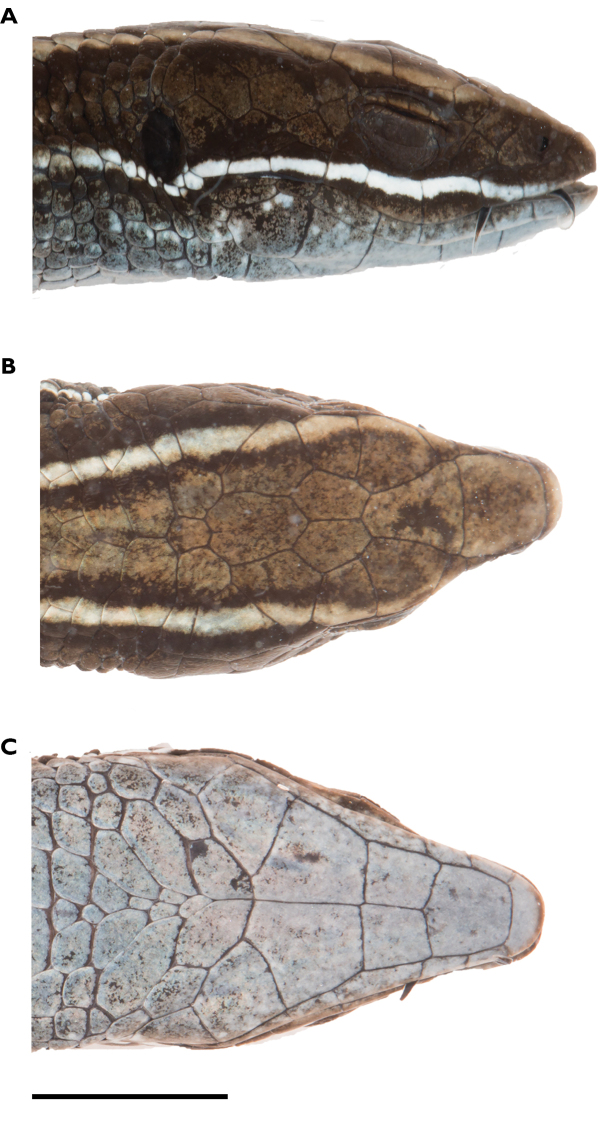
Head of holotype of *Pholidobolus condor* sp. nov. (QCAZ 15844) in lateral (**A**), dorsal (**B**), and ventral (**C)** views. Photographs by Valeria Chasiluisa. Scale bar: 5 mm.

Figures 9 , 10 Proposed standard English name: Condor cuilanes Proposed standard Spanish name: Cuilanes del Cóndor

#### Holotype.

QCAZ 15844 (Figs 9, 10), adult male, Ecuador, Provincia Morona Santiago, buffer zone of El Quimi Biological Reserve, plateau on the eastern side of El Quimi river valley, 3.51892S, 78.3690W, WGS84, 2209 m, 11 July 2017, collected by Diego Almeida, Darwin Núñez, Eloy Nusirquia, Alex Achig and Ricardo Gavilanes.

#### Paratypes (3).

Ecuador: Provincia Morona Santiago: QCAZ 16790 (hatchling), El Quimi Biological Reserve, base camp towards old heliport (high zone), 3.51894S, 78.36897W, WGS84, 2226 m, 17 April 2018; QCAZ 16788–89 (hatchlings), El Quimi Biological Reserve, near base camp, 3.5182S, 78.3913W, WGS84, 1994 m, 12 April 2018, collected by Diego Almeida, Darwin Núñez, Eloy Nusirquia, Alex Achig and María del Mar Moretta.

#### Diagnosis.

*Pholidobolus condor* is unique among its congeners, except *P. samek* sp. nov., in having green dorsolateral stripes on the head. However, adult males of *P. condor* differ from those of *P. samek* sp. nov. in having lighter dorsolateral head stripes, and reddish flanks and venter. In addition, *P. ulisesi*, *P. dicrus*, *P. hillisi*, and *P. vertebralis* differ from *P. condor* (character states of *P. condor* in parentheses) in having a conspicuous light vertebral stripe (light vertebral stripe absent). *Pholidobolus affinis*, *P. prefrontalis*, *P. dicrus*, *P. hillisi*, and *P. vertebralis* further differ from *P. condor* in having prefrontal scales (prefrontal scales absent). Additionally, *P. condor* sp. nov. has fewer dorsal scales (26−30) than *P. affinis* (45−55), *P. montium* (35−50), *P. prefrontalis* (37−46), *P. macbrydei* (31−43), and *P. dolichoderes* sp. nov. (35−40). *Pholidobolus condor* can be further distinguished from *P. fascinatus* sp. nov. by having widened medial scales on collar, and from *P. dolichoderes* sp. nov. by having fewer temporals (7–9 and 4–5, respectively), fewer ventrals (18–20 and 25–27), and fewer gulars (14–16 and 22–23).

#### Characterization.

(1) Two (rarely three) supraoculars, anteriormost larger than posterior one; (2) prefrontals absent; (3) femoral pores absent; (4) four opaque lower eyelid scales; (5) scales on dorsal surface of neck striated or smooth, progressively striated from forelimbs to tail; (6) two rows of lateral granules at midbody; (7) 27−31 dorsal scales between occipital and posterior margin of hindlimb; (8) lateral body fold present; (9) keeled ventrolateral scales on each side absent; (10) dorsum dark brown with a narrow, pale brown stripe; (11) labial stripe white; (12) flanks of body dark brown or gray; (13) hemipenial body cylindrical with distinctive capitular groove.

#### Description of holotype.

Adult male (QCAZ 15844) (Figs 9, 10); SVL 42.7 mm; TL 74.8 mm; dorsal and lateral head scales juxtaposed, finely wrinkled; rostral hexagonal, 1.67 times as wide as high; frontonasal quadrangular, slightly bigger than frontal, laterally in contact with nasal, loreal and first superciliary; prefrontal scales absent; frontal pentagonal, longer than wide, wider anteriorly, in contact with first superciliary and supraocular; frontoparietals hexagonal, longer than wide, slightly wider in the middle, each in contact laterally with supraocular II; interparietal octagonal, with a short medial suture posteriorly, lateral borders nearly parallel to each other; parietals larger than interparietal, hexagonal and positioned anterolaterally to interparietal, each in contact laterally with supraocular II and dorsalmost postocular; postparietals three, medial scale smaller than lateral ones; eight supralabials, fourth one longest and below center of eye; six infralabials, fourth one below center of eye; temporals enlarged, irregularly hexagonal, smooth; two large and smooth supratemporals; nasal shield slightly divided above nostril, irregularly pentagonal, longer than high, in contact with rostral anteriorly, first and second supralabials ventrally, frontonasal dorsally, loreal posterodorsally and frenocular posteroventrally; nostril on ventral aspect of nasal, directed laterally; loreal quadrangular, slightly wider dorsally, not in contact with supralabials; frenocular higher than long, in contact with nasal; nasal separating loreal from supralabials; two supraoculars, anteriormost one the widest; four elongate superciliaries, anteriormost enlarged, in contact with loreal; palpebral disc divided into five pigmented scales; four suboculars, anteriormost three elongated and homogeneous in size, posteriormost widest; two postoculars, the dorsalmost wider than the other; ear opening vertically oval, without denticulate margins; tympanum recessed into a shallow auditory meatus; mental wider than long; postmental pentagonal, slightly wider than long, followed posteriorly by three pairs of genials, the anterior two pairs in contact medially and the third pair separated by postgenials; all genials in contact with infralabials; gulars imbricate, smooth, widened in two longitudinal rows; posterior row of gulars (collar) with nine scales, the medial three slightly widened.

Nuchal scales slightly smaller than dorsals, except for the anteriormost that are widened; scales on sides of neck small and granular; dorsal scales elongate, imbricate, arranged in transverse rows; scales on dorsal surface of neck striated, becoming progressively keeled from forelimbs to tail; dorsal scales between occipital and posterior margin of hindlimbs 27; dorsal scale rows in a transverse line at midbody 27; one longitudinal row of smooth, enlarged ventrolateral scales on each side; dorsals separated from ventrals by two rows of small scales at the level of 13^th^ row of ventrals; lateral body fold between fore and hindlimbs present; ventrals smooth, wider than long, arranged in 20 transverse rows between collar fold and preanals; six ventral scales in a transverse row at midbody; subcaudals smooth; axillary region with granular scales; scales on dorsal surface of forelimb striated, imbricate; scales on ventral surface of forelimb granular; two thick, smooth thenar scales; supradigitals (left/right) 3/3 on finger I, 6/6 on II, 8/8 on III, 9/9 on IV, 6/6 on V; supradigitals 3/3 on toe I, 6/6 on II, 9/9 on III, 12/12 on IV, 7/7 on V; subdigital lamellae of finger I, II, III, and V single, on finger IV few scales in the middle paired; subdigital lamellae 6/6 on finger I, 11/11 on II, 15/15 on III, 17/16 on IV, 10/10 on V; subdigital lamellae on toes I and II single, on toes III, IV and V paired, except for two or three distalmost subdigitals; subdigital lamellae 7/6 on toe I, 12/12 on II, 15/16 on III, 22/22 on IV, 12/12 on V; groin region with small, juxtaposed scales; scales on dorsal surface of hindlimbs striated and imbricate; scales on ventral surface of hindlimbs smooth; scales on posterior surface of hindlimbs granular; femoral pores absent; preanal pores absent; cloacal plate paired, bordered by four scales anteriorly, of which the two medialmost are enlarged.

Additional measurements (mm) and proportions of the holotype: HL 11.0; HW 6.6; ShL 5.8; AGD 20.4; TL/SVL 1.7; HL/SVL 0.3; HW/SVL 0.2; ShL/SVL 0.1; AGD/SVL 0.5.

#### Color of holotype in life.

Dorsal background from head to base of tail dark brown, with a golden brown vertebral stripe extending from occiput to tail; greenish cream dorsolateral stripes on head, becoming light brown on posterior part of body; white longitudinal stripe extending from first supralabial to shoulder; sides of neck, flanks and limbs dark brown; chocolate brown narrow stripe extending from tympanum to arm insertion; ventrolateral region of body grayish brown; throat reddish cream; chest, belly, base of tail and lateral region of tail bright orange, with brown marks in some scales; ventral surface of hind limbs with orange diffuse marks (Fig. 9B,C).

#### Color of holotype in preservative.

Dorsal background uniformly dark brown with a grayish brown middorsal stripe extending from occiput onto tail; dorsolateral stripe distinct, pale gray, extending from snout to near base of tail; head brown dorsally (rostral, frontonasal, frontal, frontoparietals and supraoculars) and dark brown laterally; white longitudinal stripe extending from first supralabial to forelimb; lateral aspect of neck dark brown with a dorsolateral light brown stripe extending posteriorly along flanks to hindlimbs; flanks grayish brown; tail dark brown dorsally and bronze laterally; ventral surface of head gray, with dirty cream genials and scattered black marks; chest, belly and ventral surface of tail light gray with light red spots; ventral surface of limbs dark gray (Fig. 9A).

#### Variations.

Measurements and scale counts of *Pholidobolus condor* are presented in Table 6. Supraoculars three on left side in specimen QCAZ 16789; supralabials six in QCAZ 16789 and 16790, and seven in QCAZ 16788; two quadrangular frontonasals in QCAZ 16788; transverse rows of ventral scales between collar fold and preanals 18 in QCAZ 16788 and 19 in QCAZ 16790. Hatchlings with eight (QCAZ 16788–89) or six (QCAZ 16790) posterior gular (collar) scales. Unlike the adult male, hatchlings lack reddish color on tail.

**Table 6.** Summary of morphological characters and measurements (mm) of *Pholidobolus samek* sp. nov., *P. condor* sp. nov., *P. dolichoderes* sp. nov., and *P. fascinatus* sp. nov. Range (first line) and mean ± standard deviation (second line) are presented.

Character	*P. samek* sp. nov. N = 7 (adults = 3)	*P. condor* sp. nov. N = 4 (adults = 1)	*P. dolichoderes* sp. nov. N = 5 (adults = 3)	*P. fascinatus* sp. nov. N = 27 (adults = 4)
Scales along margin of upper jaw	7–10 (9.14 ± 1.07)	8–9 (8.75 ± 0.5)	9–11 (10.2 ± 0.84)	7–10 (8.36 ± 0.91)
Scales along margin of lower jaw	8–9 (8.25 ± 0.5)	5–10 (7.14 ± 2.67)	10–11 (10.2 ± 0.45)	4–10 (7.4 ± 1.58)
Gulars	15–18 (16.71 ± 1.11)	14–16 (15 ± 0.82)	22–23 (22.8 ± 0.48)	14–17 (15.72 ± 0.89)
Ventrals in transverse row at midbody	19–21 (20 ± 0.82)	18–20 (19 ± 1.15)	25–27 (25.8 ± 0.84)	21–25 (22.96 ± 1.21)
Dorsals from occiput to base of tail	27–29 (27.71 ± 0.76)	26–30 (27.75 ± 1.71)	35–40 (36.8 ± 2.05)	32–37 (34.64 ± 1.19)
Temporals	4–5 (4.14 ± 0.38)	4–5 (4.25 ± 0.5)	7–9 (8 ± 0.70)	3–5 (3.44 ± 0.65)
Scales around midbody	25–32 (27.71 ± 2.75)	27–30 (28 ± 1.41)	31–33 (32.2 ± 0.84)	28–34 (30.96 ± 1.79)
Scales around tail	14–16 (15 ± 0.81)	14–20 (17.86 ± 2.73)	18–19 (18.6 ± 0.55)	18–22 (20.32 ± 1.18)
Lower eyelid scales	4–5 (4.14 ± 0.38)	5	4–6 (4.8 ± 0.84)	4–6 (5.04 ± 0.61)
Gular (collar) scales	6–8 (7.14 ± 0.9)	6–9 (7.75 ± 1.26)	6–8 (6.4 ± 0.89)	9–12 (10.28 ± 0.73)
Head length in adults	9.9–11.4 (10.76 ± 0.77)	11	9.7–10.6 (10.05 ± 0.46)	8.9–12.3 (10.22 ± 1.80)
Head width in adults	6.5–7.4 (6.93 ± 0.48)	6.6	6.2–6.3 (6.26 ± 0.05)	6.6–9.2 (7.58 ± 1.45)
SVL in adults	41.6–49.3 (45.89 ± 3.89)	42.7	41.1–50.6 (45.75 ± 4.74)	42.6–52.5 (47.3 ± 4.98)

#### Distribution and natural history.

**Figure 11.**
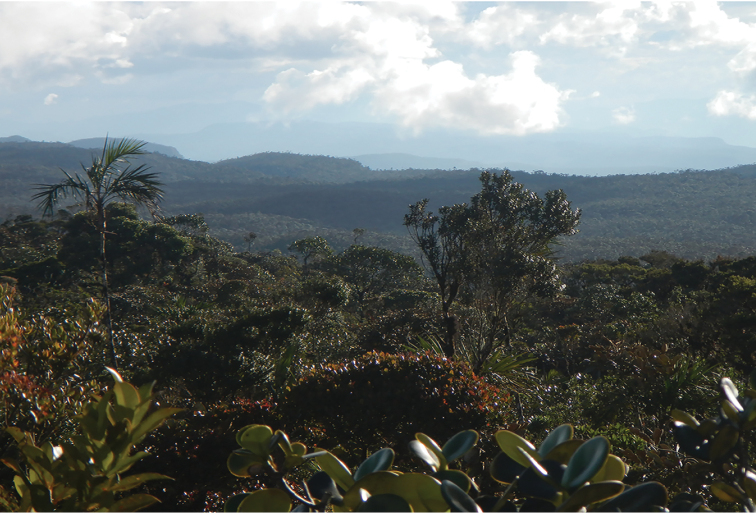
Habitat of *Pholidobolus condor* sp. nov. at El Quimi Biological Reserve, Ecuador. Photographs by Álvaro Pérez.

*Pholidobolus condor* occurs in Cordillera del Cóndor in southeastern Ecuador at elevations between 1994–2226 m. The new species is known from El Quimi Biological Reserve in Morona Santiago province (Fig. 7). The holotype was found active at 21h14 at the base of a bromeliad on a sandstone plateau of shrub vegetation (Fig. 11).

Several eggs were found within a bromeliad, suggesting that females of *P. condor* lay their eggs in communal nests. Four eggs that were found on the ground at the base of bromeliads and under a trunk were incubated in sphagnum and perlite in captivity for approximately three months. On average, hatchlings weighted 0.4 g and were 23.7 mm in SVL.

#### Conservation status.

*Pholidobolus condor* is only known from Cordillera del Cóndor in southeastern Ecuador. This area is currently threatened by mining activities (Ron et al. 2018; Valencia et al. 2017; Van Teijlingen 2016). Habitat destruction and fragmentation is evident at a distance of ~11 km from the collection sites (Mazabanda et al. 2018). Because of the small known distribution and habitat disturbance, we suggest assigning *P. condor* to the Critically Endangered category under criteria B1a, b(iii); C1; D, according to IUCN (2012) guidelines.

#### Etymology.

The specific epithet *condor* refers to Cordillera del Cóndor, where the new species was discovered. The Cordillera del Cóndor is an eastern outlier of the main Andean chain, where a significant number of species have been discovered in the last decade (Brito et al. 2017; Huamantupa-Chuquimaco and Neill 2018; Ron et al. 2018; Torres-Carvajal et al. 2009; Valencia et al. 2017).

#### Remarks.

See remarks on *Pholidobolus samek* sp. nov. above.

### Pholidobolus dolichoderes sp. nov.

Taxon classificationAnimaliaSquamata Gymnophthalmidae

9B751BAF-386E-5BAC-8E33-48F8E8C21992

http://zoobank.org/95D82201-D761-40F4-8395-4AAC38F24563

**Figure 12.**
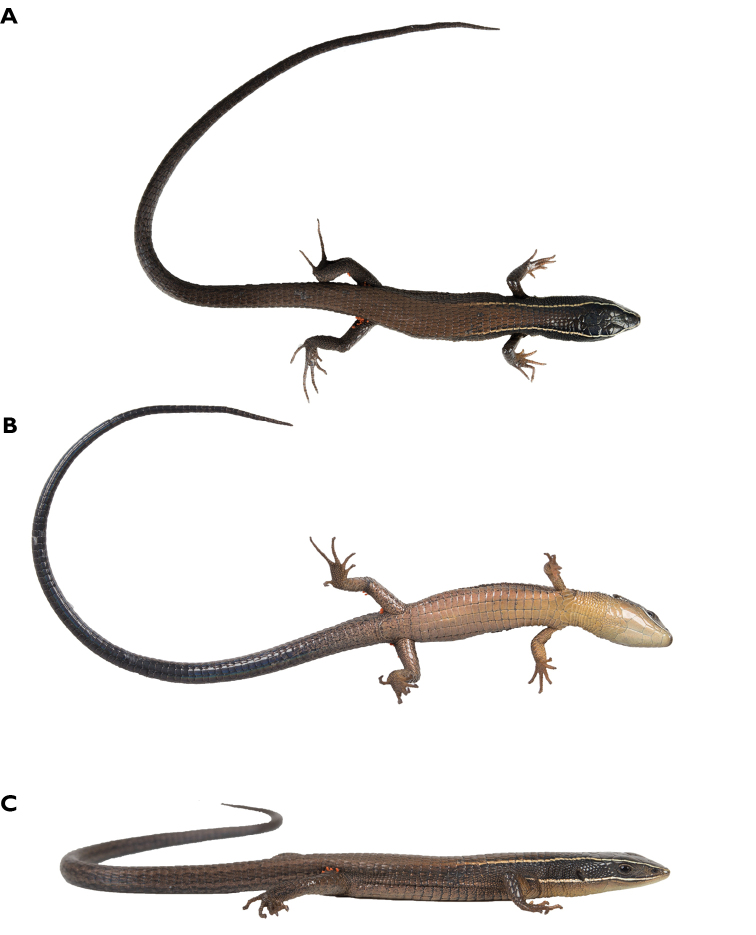
Holotype of *Pholidobolus dolichoderes* sp. nov. (QCAZ 16353) in life in dorsal (**A**), ventral (**B**), and lateral (**C**) views. Male, SVL = 41.1 mm. Photographs by Gustavo Pazmiño.

**Figure 13.**
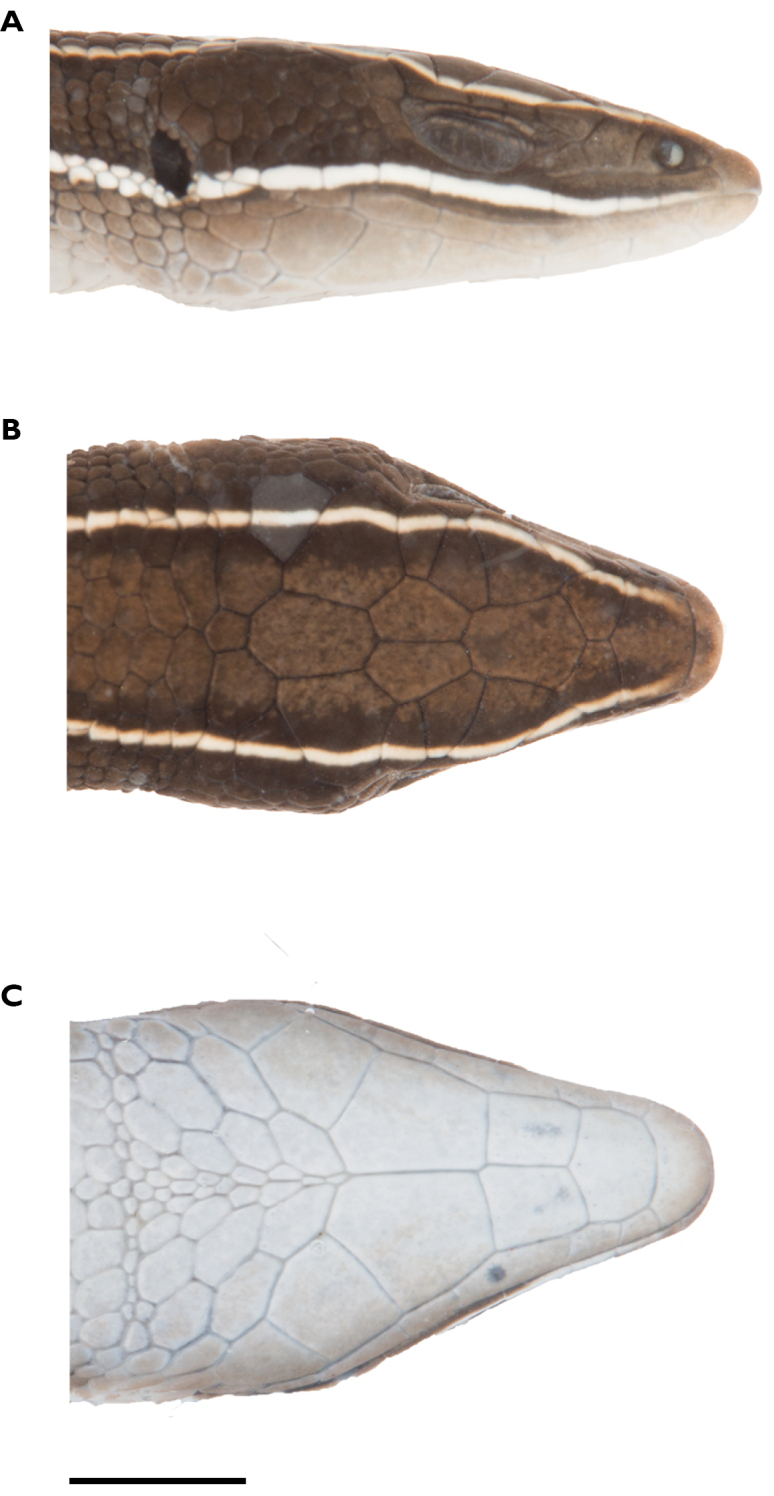
Head of holotype of *Pholidobolus dolichoderes* sp. nov. (QCAZ 16353) in lateral (**A**), dorsal (**B**), and ventral (**C**) views. Photographs by Valeria Chasiluisa. Scale bar: 5 mm.

**Figure 14.**
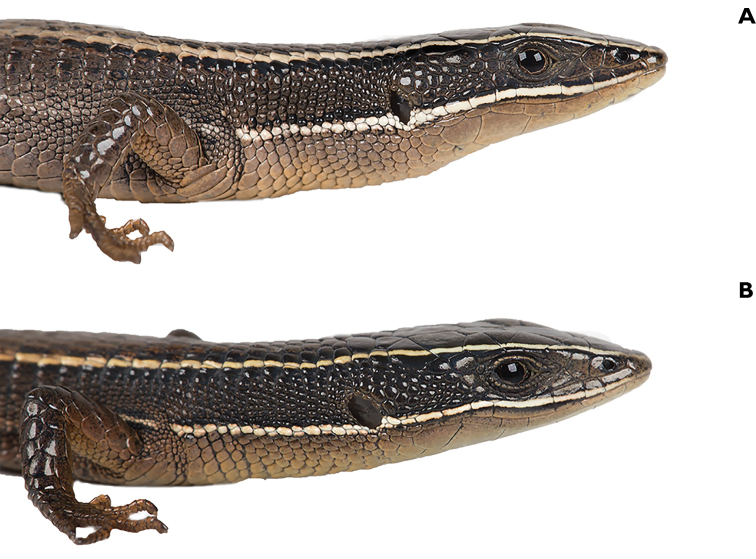
Close-up of head and neck of *Pholidobolus dolichoderes* sp. nov. in life. QCAZ 16349 (**A** adult female); QCAZ 16353 (**B** male holotype). Photographs by Gustavo Pazmiño.

Figures 12 , 13 , 14 Proposed standard English name: Long-necked cuilanes Proposed standard Spanish name: Cuilanes de cuello largo

#### Holotype.

QCAZ 16353 (Figs 12, 13), adult male, Ecuador, Provincia Azuay, San Felipe de Oña, 3.4292S, 79.2364W, WGS84, 2672 m, 16 March 2018, collected by Diego Almeida, Darwin Núñez, Eloy Nusirquia, Alex Achig and Katherine Nicolalde.

#### Paratypes (4).

Ecuador: Provincia Azuay: QCAZ 16349, 16352 (adult females), San Felipe de Oña, Susudel-Poetate road, 3.4322S, 79.2369W, WGS84, 2506 m, 16 March 2018; QCAZ 16350–51 (juveniles), San Felipe de Oña, 3.4275S, 79.2339W, WGS84, 2675 m, 16 March 2018, same collectors as holotype.

#### Diagnosis.

*Pholidobolus dolichoderes* is unique among its congeners in having a long neck with granular scales between the posterior corner of the orbit and the anterior edge of the tympanum, as well as an inconspicuous ventrolateral fold between fore and hindlimbs. In addition, *P. ulisesi*, *P. dicrus*, *P. hillisi*, and *P. vertebralis* differ from *P. dolichoderes* in having a conspicuous light vertebral stripe. The new species further differs from *P. affinis* in lacking ocelli on flanks, and from *P. condor* sp. nov., *P. macbrydei*, and *P. montium* in having prefrontal scales. *Pholidobolus dolichoderes* has more dorsals (35–40) and ventrals (25–27) than *P. samek* sp. nov. (27–29 and 19–21, respectively) and *P. condor* sp. nov. (26–30 and 18–20), and, unlike *P. fascinatus* sp. nov., it has widened medial scales on collar. In addition, *P. dolichoderes* has more temporals (7–9) and gulars (22–23) than *P. samek* sp. nov. (4–5 and 15–18, respectively), *P. condor* sp. nov. (4–5 and 14–16), and *P. fascinatus* sp. nov. (3–5 and 14–17).

#### Characterization.

(1) Three supraoculars, anteriormost larger than posterior ones; (2) prefrontals present; (3) femoral pores present in both sexes; (4) four to six opaque lower eyelid scales; (5) scales on dorsal surface of neck smooth, becoming slightly keeled from forelimbs to tail; (6) two or three rows of lateral granules at midbody; (7) 35−20 dorsal scales between occipital and posterior margin of hindlimb; (8) lateral body fold present but inconspicuous; (9) keeled ventrolateral scales on each side absent; (10) dorsum dark brown with a diffuse pale brown vertebral stripe that becomes grayish brown towards tail; (11) labial stripe white; (12) flanks of body gray brown; (13) white stripe along forelimb present; (14) hemipenial body cylindrical, with sulcus spermaticus originating between thick lips.

#### Description of holotype.

Adult male (QCAZ 16353) (Figs 12, 13); SVL 41.1 mm; TL 96.3 mm; dorsal and lateral head scales imbricated, smooth; rostral hexagonal, 1.75 times as wide as high; frontonasal heptagonal, slightly wider than long, laterally in contact with nasal, similar in size to frontal; prefrontals present, in wide contact medially, and in contact with loreal and first superciliary laterally; frontal hexagonal, longer than wide, wider anteriorly, in contact with first and second supraoculars; frontoparietals hexagonal, longer than wide, slightly wider posteriorly, each in contact with second and third supraoculars, parietals and interparietal; interparietal heptagonal, lateral borders nearly parallel to each other; parietals wider than interparietal, heptagonal, and positioned anterolaterally to interparietal, each in contact with third supraocular and dorsalmost postocular; postparietals three, medial scale smaller than lateral ones; seven supralabials, fourth one the longest and below center of eye; five infralabials, fourth one below center of eye; temporals small, irregularly, smooth; supratemporal scales not well differentiated, smooth; nasal shield divided above the nostril, longer than high, in contact with rostral anteriorly, first and second supralabials ventrally, frontonasal dorsally, loreal posteriorly; loreal pentagonal, slightly wider dorsally, in contact with second and third supralabials; frenocular longer than high, in contact with loreal; three supraoculars, with the first one being the widest; four elongate superciliaries, anteriormost one enlarged, in contact with loreal; palpebral disc oval, pigmented, divided into four scales; four suboculars, two elongated and similar in size, the anteriormost and posteriormost larger than the others; three postoculars, dorsalmost wider than the others; ear opening vertically oval, without denticulate margins; tympanum recessed into a shallow auditory meatus; mental wider than long; postmental pentagonal, slightly wider than long, followed posteriorly by three pairs of genials, the anterior two pairs in contact medially and the third pair separated by postgenials; all genials in contact with infralabials; gulars imbricate, smooth, widened in two longitudinal rows; gular fold complete, posterior row of gulars (collar) with six scales, the medial two distinctly widened.

Nuchal scales slightly smaller than dorsals, except for the anteriormost that are widened; scales on sides of neck small and granular; dorsal scales elongate, juxtaposed, arranged in transverse rows; scales on dorsal surface of neck striated, becoming slightly keeled from forelimbs to tail; dorsal scales between occipital and posterior margin of hindlimbs 35; dorsal scale rows in a transverse line at midbody 32; one longitudinal row of smooth, enlarged ventrolateral scales on each side; dorsals separated from ventrals by three rows of granular scales at level of 13^th^ row of ventrals; lateral body fold between fore and hindlimbs poorly defined; ventrals smooth, arranged in 26 transverse rows between collar fold and preanals; six ventral scales in a transverse row at midbody; subcaudals smooth; axillary region with granular scales; scales on dorsal surface of forelimb smooth, imbricate; scales on ventral surface of forelimb granular; two thick, smooth thenar scales; supradigitals (left/right) 3/0 on finger I, 7/7 on II, 9/8 on III, 10/10 on IV, 5/5 on V; supradigitals 4/4 on toe I, 7/7 on II, 11/11 on III, 12/11 on IV, 9/8 on V; subdigital lamellae of fingers I and II mostly single, III and IV paired proximally, on finger V all single; subdigital lamellae 5 on left finger I (right finger missing), 10/10 on II, 14/14 on III, 14/14 on IV, 9/9 on V; subdigital lamellae on toe I single, on toe II paired at the middle, on toe III and IV paired along proximal half, and on toe V paired proximally; subdigital lamellae 5/5 on toe I, 10/10 on II, 14/14 on III, 18/19 on IV, 11/11 on V; groin region with small, imbricate scales; scales on dorsal surface of hindlimbs striated and imbricate; scales on ventral surface of hindlimbs smooth; scales on posterior surface of hindlimbs granular; femoral pores present, three on left leg and five on right leg; preanal pores absent; cloacal plate paired, bordered by four scales anteriorly, of which the two medialmost are enlarged.

Additional measurements (mm) and proportions of the holotype: HL 9.8; HW 6.2; ShL 5.4; AGD 20.7; TL/SVL 2.4; HL/SVL 0.2; HW/SVL 0.1; ShL/SVL 0.1; AGD/SVL 0.5.

#### Color of holotype in life.

Dorsal background of head dark brown; diffuse pale brown vertebral stripe that becomes grayish brown towards tail; creamy white dorsolateral stripes on head extending posteriorly and fading away at midbody; white longitudinal stripe extending from first supralabial to shoulder; sides of neck brown; flanks grayish brown with diffuse dark brown marks; limbs brown; ventrolateral region of body grayish brown; throat and chest cream; belly grayish cream; base of tail gray with dark little spots (Figs 12, 14B).

#### Color of holotype in preservative.

Dorsal background uniformly brown with a diffuse light brown vertebral stripe extending from occiput onto tail, but fading at posterior end of body; dorsal and ventral surface of head brown; flanks light brown, with scattered dark brown spots; head and neck with two distinct white longitudinal stripes, the ventral one extending from first supralabial to forelimb, and the dorsal one from canthus rostralis to scapular region, posterior to which if fades into a light brown stripe; lateral aspect of neck dark brown; tail grayish brown; gular, chest and venter regions pale gray; ventral surface of tail and limbs gray.

#### Variations.

Measurements and scutellation data of *Pholidobolus dolichoderes* are presented in Table 6. Superciliaries 4/5 (left/right) in specimen QCAZ 16350; palpebral disc divided into 5/6 scales in QCAZ 16352 and 3/5 in QCAZ 16351; frontonasal pentagonal in QCAZ 16349–52; prefrontals pentagonal in QCAZ 16349, 16350 and 16352; two rows of lateral granules at midbody in QCAZ 16439, 16350 and 16351. Usually six gular (collar) scales, eight in QCAZ 16349. Male is smaller (SVL 41.1 mm, *N* = 1) than females (maximum SVL 48.1 mm, *N* = 2).

Adult females differ from holotype in having a grayish brown vertebral stripe, fading away posteriorly, and grayish brown flanks (Fig. 14). Juvenile QCAZ 16350 differs from holotype in having grayish brown flanks, without scattered dark brown spots; juvenile QCAZ 16351 is unique in having white spots on flanks and over forelimbs.

#### Distribution and natural history.

*Pholidobolus dolichoderes* is known to occur between 2506−2675 m in San Felipe de Oña, southwestern Azuay province (Fig. 7). This area is composed of many different landscapes including small valleys, desert areas and wet paramo. Most specimens were found active at day (10h26–15h30), mostly on the ground or near spiny ground bromeliads known as achupallas (*Puya* sp.).

#### Conservation status.

*Pholidobolus dolichoderes* is only known from unprotected localities around Oña. The population size of this species is unknown, but our sampling suggests low abundances. Because of the small known distribution and lack of additional data, we suggest assigning *P. dolichoderes* to the Data Deficient category according to IUCN (2012) guidelines.

#### Etymology.

The specific epithet *dolichoderes* derives from the Greek words *dolikhós*, meaning long, and *derē*, meaning neck, in allusion to the distinctively long neck of this species.

### Pholidobolus fascinatus sp. nov.

Taxon classificationAnimaliaSquamata Gymnophthalmidae

CF0A8696-EA82-5FD6-9E90-121871AD3950

http://zoobank.org/C5EC3F40-41DF-4A7D-A3AA-C2C8A6EF81C1

**Figure 15.**
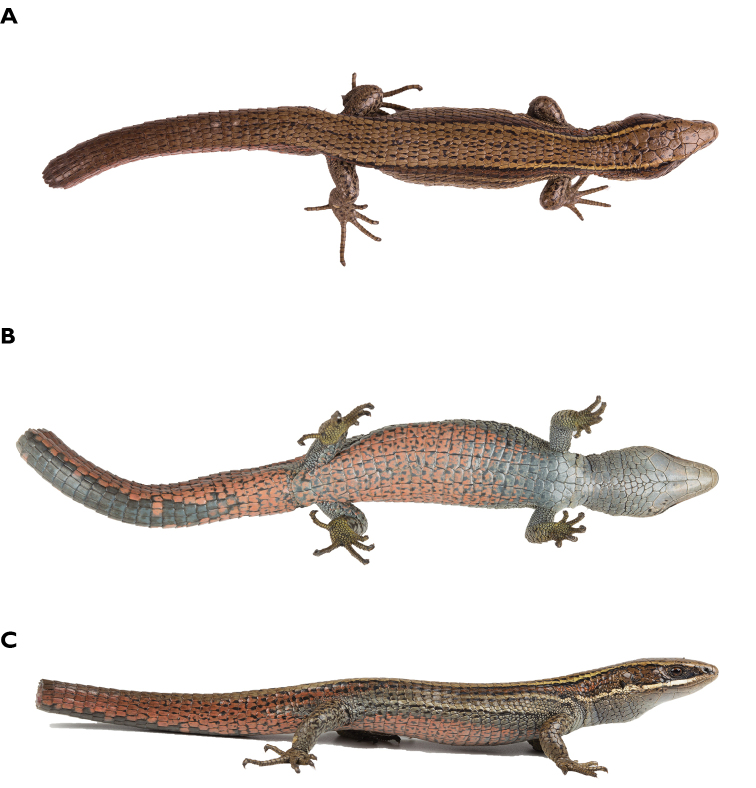
Holotype of *Pholidobolus fascinatus* sp. nov. (QCAZ 15120) in life in dorsal (**A**), ventral (**B**), and lateral (**C**) views. Male, SVL = 52.5 mm. Photographs by Diego Quirola.

**Figure 16.**
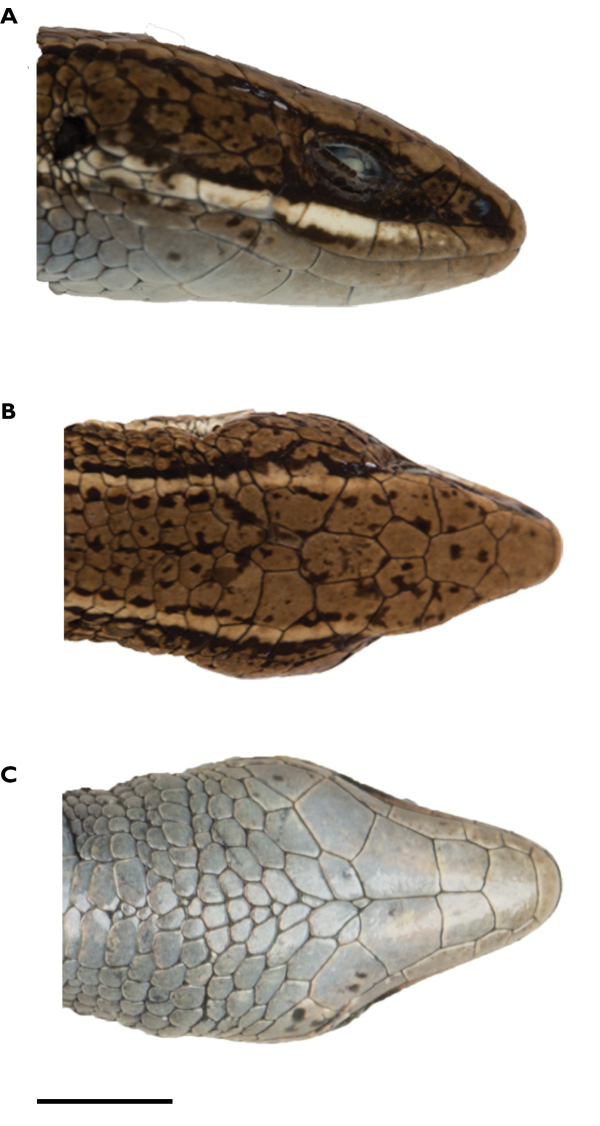
Head of holotype of *Pholidobolus fascinatus* sp. nov. (QCAZ 15120) in lateral (**A**), dorsal (**B**), and ventral (**C**) views. Photographs by Valeria Chasiluisa. Scale bar: 5 mm.

**Figure 17.**
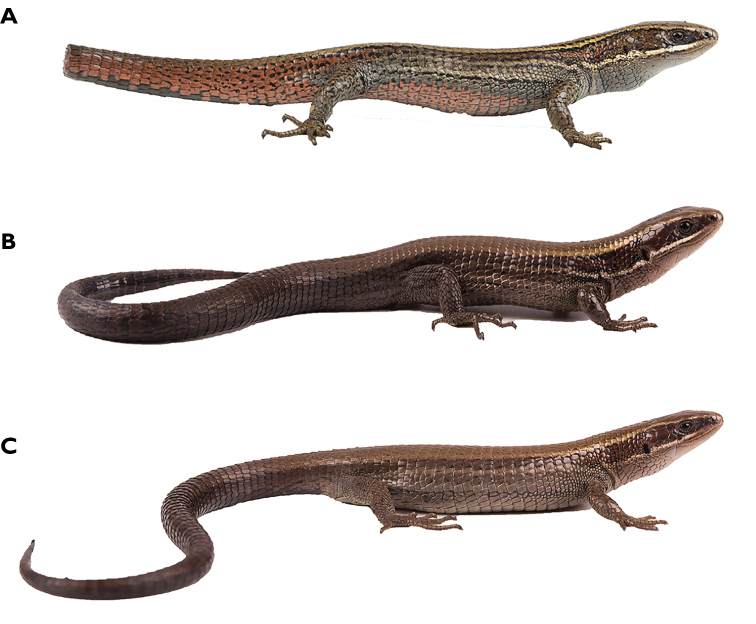
Color variation in live specimens of *Pholidobolus fascinatus* sp. nov. **A** male holotype (QCAZ 15120, SVL = 52.5 mm) **B** male paratype (QCAZ 15122, SVL = 42.6 mm) **C** female paratype (QCAZ 15118, SVL = 46.7 mm).

Figures 15 , 16 , 17 Proposed standard English name: Haunted cuilanes Proposed standard Spanish name: Cuilanes encantados

#### Holotype.

QCAZ 15120 (Figs 15, 16), adult male, Ecuador, Provincia El Oro, Lake Chillacocha, 3.4984S, 79.6188W, WGS84, 3382 m, 20 November 2016, collected by Diego Almeida, Darwin Núñez, Eloy Nusirquia, Santiago Guamán and Guadalupe Calle.

#### Paratypes (26).

Ecuador: Provincia El Oro: QCAZ 15122 (adult male), QCAZ 15121 (adult female), QCAZ 15169−73, 15177−78, 15180, 15193, 15221, 15243−44, 15396−15405 (juveniles), same data as holotype; QCAZ 15118 (adult female), Lake Chillacocha, 3.4986S, 79.6187W, WGS84, 3348 m, 17 November 2016, same collectors as holotype.

#### Diagnosis.

*Pholidobolus fascinatus* is unique among its congeners in lacking widened medial scales on collar (posterior row of gulars). In addition, *P. fascinatus* differs from *P. affinis*, *P. prefrontalis*, *P. macbrydei*, *P. dolichoderes* sp. nov., and *P. montium* in having a loreal scale frequently in contact with the supralabials (loreal scale, if present, not in contact with supralabials in the other species). *Pholidobolus ulisesi*, *P. dicrus*, *P. hillisi*, *P. paramuno*, and *P. vertebralis* differ from *P. fascinatus* in having a conspicuous light vertebral stripe. *Pholidobolus samek* sp. nov. and *P. condor* sp. nov. differ from *P. fascinatus* in having bright green dorsolateral stripes on the head. In addition, *P. fascinatus* has more dorsals (32–37) and ventrals (21–25) than *P. samek* sp. nov. (27–29 and 19–21, respectively) and *P. condor* sp. nov. (26–30 and 18–20); and it has fewer temporals (3–5) and gulars (14–17) than *P. dolichoderes* sp. nov. (7–9 and 22–23, respectively).

#### Characterization.

(1) Two (rarely three) supraoculars, anteriormost larger than posterior one; (2) prefrontals present or absent; (3) femoral pores absent in both sexes; (4) four to six opaque lower eyelid scales; (5) scales on dorsal surface of neck smooth, becoming striated from forelimbs to tail; (6) one row of lateral granules at midbody; (7) 32–37 dorsal scales between occipital and posterior margin of hindlimb; (8) lateral body fold present; (9) dorsum brown with a diffused chocolate brown middorsal stripe that fades away towards tail; (11) labial stripe white or cream; (12) flanks of body brown; (13) conical hemipenial body, with sulcus spermaticus originating between distinctly thick lips; (14) 22 flounces extending along hemipenial body.

#### Description of holotype.

Adult male (QCAZ 15120) (Figs 15, 16); SVL 52.5 mm; TL 37.6 mm; dorsal and lateral head scales juxtaposed, finely wrinkled; rostral hexagonal, 2.27 times as wide as high; frontonasal hexagonal, wider than long, in contact with nasal laterally, slightly larger than frontal; prefrontal scales irregularly pentagonal; frontal heptagonal, longer than wide, slightly wider anteriorly, in contact with prefrontals and frontonasal anteriorly, two supraoculars laterally, and frontoparietals posteriorly; frontoparietals pentagonal, longer than wide, slightly wider posteriorly, each in contact laterally with supraocular II; interparietal heptagonal, lateral borders nearly parallel to each other; parietals hexagonal, each in contact laterally with supraocular II and dorsalmost postocular; postparietals four, with medial scales less than half the size of lateral ones; eight supralabials, fourth one the longest and below center of eye; eight infralabials, third and fourth one below center of eye; temporals enlarged, irregularly hexagonal, juxtaposed, smooth; two large, smooth supratemporal scales; nasal divided, irregularly pentagonal, longer than high, in contact with rostral anteriorly, first and second supralabials ventrally, frontonasal dorsally, loreal posterodorsally and frenocular posteroventrally; nostril in center of nasal, directed lateroposteriorly; loreal rectangular, wider ventrally; frenocular longer than high, higher anteriorly, in contact with nasal, separating loreal from supralabials; two supraoculars, homogeneous in size; four superciliaries, anteriormost enlarged and in contact with loreal; palpebral disc divided into five pigmented scales; suboculars elongated, four on right side and three on left side; two postoculars, dorsalmost wider than the other; ear opening vertically oval, without denticulate margins; tympanum recessed into a shallow auditory meatus; mental semicircular, longer than wide; postmental pentagonal, slightly longer than wide, followed posteriorly by three pairs of genials, the anterior two in contact medially and the posterior one separated by postgenials; all genials in contact with infralabials; gulars imbricate, smooth, widened in two longitudinal rows; posterior row of gulars (collar) with 11 scales that are similar in size.

Nuchal scales similar in size to dorsals, except for the anteriormost that are widened; scales on sides of neck small and slightly granular; dorsal scales hexagonal, elongate, imbricate, arranged in transverse rows; scales on dorsal surface of neck smooth, becoming progressively striated from forelimbs to tail; dorsal scales between occipital and posterior margin of hindlimbs 33; dorsal scale rows in a transverse line at midbody 25; dorsals separated from ventrals by one row of small scales at level of 13^th^ row of ventrals; lateral body fold between fore and hindlimbs present; ventrals smooth, wider than long, arranged in 25 transverse rows between collar fold and preanals; six ventral scales in a transverse row at midbody; subcaudals smooth; axillary region with granular scales; scales on dorsal surface of forelimb striated, imbricate; scales on ventral surface of forelimb granular; two thick, smooth thenar scales; supradigitals (left/right) 3/3 on finger I, 7/6 on II, 8/8 on III, 10/10 on IV, 5/5 on V; supradigitals 3/3 on toe I, 6/6 on II, 8/9 on III, 11/11 on IV, 8/8 on V; subdigital lamellae of finger I single, on finger II all paired, except by the three distalmost, on finger III (proximal half) paired, on finger IV slightly paired at the middle, on finger V all single in right finger and three paired in left finger; subdigital lamellae 5/5 on finger I, 9/9 on II, 13/13 on III, 14/15 on IV, 9/9 on V; subdigital lamellae on toes I and II paired proximally and single distally, on toes III, IV and V paired, except for the three to five distalmost subdigitals; subdigital lamellae 5/5 on toe I, 10/10 on II, 14/13 on III, 18/18 on IV, 11/12 on V; groin region with small, imbricate scales; scales on dorsal surface of hindlimbs smooth and imbricate; scales on ventral surface of hindlimbs smooth; scales on posterior surface of hindlimbs granular; femoral pores absent; preanal pores absent; cloacal plate paired, bordered anteriorly by two enlarged scales.

Additional measurements (mm) and proportions of the holotype: HL 12.3; HW 9.2; ShL 6.7; AGD 26.5; TL/SVL 0.7; HL/SVL 0.2; HW/SVL 0.2; ShL/SVL 0.1; AGD/SVL 0.5.

#### Color in life of the holotype.

Dorsal background from head to base of tail brown, with a diffuse chocolate-brown middorsal stripe that fades away towards tail; light brown dorsolateral stripes on head extending posteriorly and fading away at midbody; white longitudinal stripe extending from third supralabial to shoulder; sides of neck, flanks, and limbs brown; reddish brown narrow stripe extending from tympanum to arm insertion; ventrolateral region of body grayish brown; throat and chest gray; belly background gray with conspicuous orange marks; tail orange anteriorly and laterally (Figs 15, 17A).

#### Color in preservative of the holotype.

Dorsal background uniformly brown with a cream brown vertebral stripe extending from head onto tail; vertebral stripe slender anteriorly, becoming slightly wider posteriorly; head light brown with black dots dorsally (rostral, frontonasal, frontal, frontoparietals and supraoculars) and brown laterally; cream longitudinal stripe extending from third supralabial to shoulder; ventrolateral aspect of neck brown; forelimbs with scattered black dots; flanks brown; tail brown dorsally; ventral surface of head light gray, chest and venter dark gray, ventral surface of tail slightly brown, with scattered dark brown marks.

#### Variations.

Measurements and scale counts of *Pholidobolus fascinatus* are presented in Table 6. Supralabials 9/9 (left/right) in specimens QCAZ 15118 and 15122, and supraoculars 3/3 in QCAZ 15118; loreal scale absent in QCAZ 15118; prefrontals absent in QCAZ 15122 and 15173; little intrusive scales between postparietal and frontoparietals in QCAZ 15118, 15121 and 15122; frontonasal quadrangular in QCAZ 15122; frontal nonagonal and pentagonal in QCAZ 15118 and 15173, respectively; interparietal hexagonal in QCAZ 15122; parietal pentagonal in QCAZ 15170. Four posterior cloacal scales in QCAZ 15118. Males are slightly smaller (SVL 47.6 mm, *N* = 2) than female (maximum SVL 48.2 mm, *N* = 2). Adult male QCAZ 15122 differs from holotype in having sides of tail and chest dark brown without gray spots. Adult female QCAZ 15118 differs from holotype in having a light gray chest, a dark gray ventral surface of tail, dark brown sides of tail, and in lacking orange or red brown color on sides of neck (Fig. 17).

#### Distribution and natural history.

*Pholidobolus fascinatus* inhabits wet paramo in the western slopes of the Andes of southern Ecuador (Fig. 7). The new species is known only from El Oro province, at 3348−3382 m. All specimens were found active at 14h00–17h00 mostly under stones.

We found 41 eggs (17 as fragmented eggshells) in a communal nest next to male QCAZ 15120. We incubated the 24 unhatched eggs in soil and perlite in captivity. They were 11.9–13.2 mm long, 5.5–9.2 mm wide, and weighted 0.5 g on average. Hatchlings (*N* = 20) weighted 0.4 g and were 26.2 mm in SVL on average.

#### Conservation status.

*Pholidobolus fascinatus* is only known from localities around Lake Chillacocha. The population size for this species is unknown, but our sampling suggests average abundances. Because of the small known distribution and lack of additional data, we suggest assigning *P. fascinatus* to the Data Deficient category, according to IUCN (2012) guidelines.

#### Etymology.

The species epithet *fascinatus* is a Latin word meaning enchanted, in allusion to Lake Chillacocha, also known as the Enchanted Lake. According to local belief, this lake is enchanted and has healing powers.

## Discussion

The systematics of *Pholidobolus* and its sister taxon *Macropholidus* have been controversial partly because morphological evidence has been misinterpreted. Nonetheless, the recent use of molecular phylogenies has reshaped the systematics and taxonomy of this clade (Torres-Carvajal and Mafla-Endara 2013; Torres-Carvajal et al. 2015). In addition, recent collections in poorly explored areas along the Andes of Colombia, Ecuador, and Peru have led to the discovery and description of new species (Hurtado-Gómez et al. 2018; Torres-Carvajal et al. 2014; Venegas et al. 2016). In this paper we use morphological and molecular evidence to describe four new species of *Pholidobolus*, all except *P. dolichoderes* sp. nov. from remote highlands, based mostly on recent collections in southern Ecuador. Unexpectedly, allocating the new species described herein within the phylogenetic tree of *Pholidobolus* rendered *P. macbrydei* paraphyletic, suggesting that populations currently assigned to this taxon represent multiple species, some of which (e.g., Clades A and F) match the evolutionary significant units identified by Mafla-Endara (2011). Nonetheless, we refrain from describing any of these putative species (Clades A–F) in this paper as we believe that further sampling and analysis are necessary. According to our PCA results, three of the four new species are morphologically different from other “*Pholidobolus macbrydei*” (Fig. 2). Components I and II in the PCA, however, explain less than 50% of the variation within the “*P. macbrydei*” clade (Table 5). Thus, it is necessary to study additional morphological characters and increase sample size to better elucidate morphological differences. Mafla-Endara (2011) also suggested hybridization between *P. macbrydei* from Cañar province and *P. prefrontalis* based on both the relatively great variation in morphology within the Cañar populations, and their morphological similarity to *P. prefrontalis*. Nevertheless, our nuclear phylogenetic tree does not suggest hybridization between *P. macbrydei* and *P. prefrontalis* (Appendix II).

Current evidence prevents us from assigning the name *P. macbrydei* to any of the recovered clades. However, we suspect that *P. macbrydei* belongs or is more closely related to Clades C, D, and E for two reasons (Fig. 1). First, adult males in these clades match closely the description of *P. macbrydei* (Montanucci 1973). Second, Clades C, D, and E lie nearby the type locality of *P. macbrydei*. It is noteworthy that Clade B also lies near the type locality of *P. macbrydei* (Fig. 7), although males in Clade B lack the red lateral stripes characteristic of *P. macbrydei*. DNA samples from the type locality should help clarify the taxonomy of this group.

The Cordillera del Cóndor is a sub-Andean mountain chain geologically similar to the Tepuis of the Guiana region. It is composed of marine and continental sediments (Neill 2005). This area is presently threatened by mining activities, despite discovery of a significant number of new species in the last ten years (Brito et al. 2017; Huamantupa-Chuquimaco and Neill 2018; Mashburn et al. 2020; Ron et al. 2018; Torres-Carvajal et al. 2009; Valencia et al. 2017) suggesting that Cordillera del Cóndor is a diversity hotspot. Our discovery of *P. samek* and *P. condor* further supports this idea. Therefore, we strongly advise authorities to improve conservation efforts for Cordillera del Cóndor.

The discovery of four new species and a paraphyletic *P. macbrydei* reveals high levels of unexpected diversity within *Pholidobolus* from southern Ecuador. This study supports the idea that Andean herpetofauna in this region is more diverse in species numbers than previously thought (Sánchez-Pacheco et al. 2012), especially for poorly explored areas like Cordillera del Cóndor. Collections in this area are usually scarce due to complex logistics. However, we recommend more intensive sampling efforts. Future studies should include larger samples and other types of evidence (e.g., genomic data, environmental variables) that might prove useful for species delimitation within *Pholidobolus* and other vertebrate taxa.

## Supplementary Material

XML Treatment for Pholidobolus samek

XML Treatment for Pholidobolus condor

XML Treatment for Pholidobolus dolichoderes

XML Treatment for Pholidobolus fascinatus

## Figures and Tables

**Figure:**
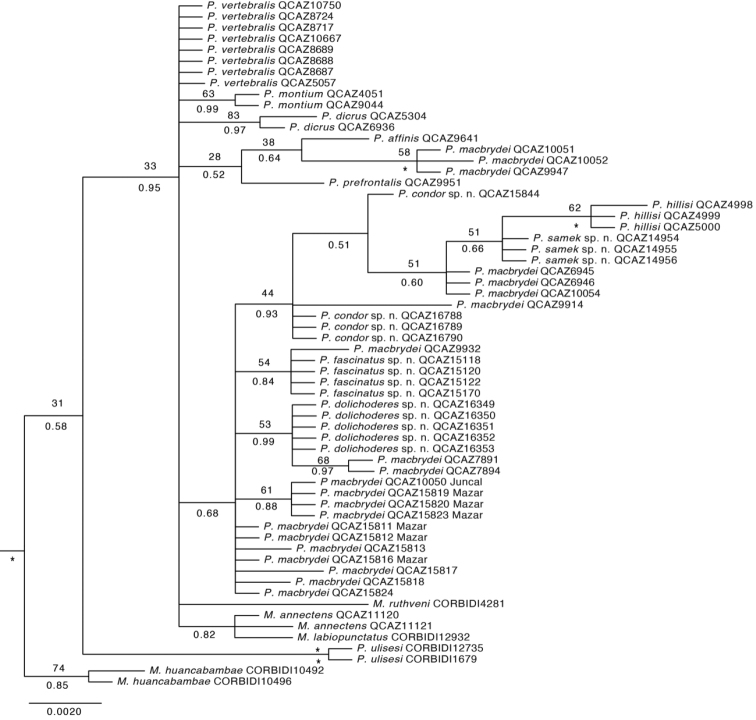
Phylogeny of *Pholidobolus*. ML phylogram derived from the analysis of 411 bp of nuclear DNA. Bootstrap values are shown above branches and Bayesian posterior probabilities below branches (≤ 50% not shown). Asterisks indicate maximum values. The outgroup taxon (*Anadia rhombifera*) is not shown. Species names followed by voucher numbers are shown.

## Appendix I

Additional specimens examined of *Pholidobolus macbrydei*. ECUADOR: **Provincia Azuay**: Cuenca-Azogues, 2.895222S, 78.95822W, 2486 m, QCAZ 6985; Cuenca-Chaucha, 2.861209S, 79.37869W, 2943 m, QCAZ 9668; Cuenca-Cochapamba, 2.797120S, 79.41562W, 3548 m, QCAZ 10133–10135; Cuenca-El Cajas, 2.77744S, 79.17001W, 3508 m, QCAZ 9932, 9936; 2.74105S, 79.23479W, 4092 m, QCAZ 8010; 2.776299S, 79.23743W, 4068 m, QCAZ 8011; 3.04155S, 79.21567W, 3766 m, QCAZ 8897, 8899–8903, 8906; Cuenca-Mazan Forest, 2.87522S, 79.12923W, 3189 m, QCAZ 8008, 8013; Cutchil, 3.133999S, 78.81300W, 2900 m, QCAZ 823; Guablid, 2.77488S, 78.69758W, 2453 m, QCAZ 9915, 9919–9920; Gualaceo, 2.909767S, 78.73436W, 2625 m, QCAZ 10875; Gualaceo-Limón; 2.948S, 78.71200W, 3110 m, QCAZ 819–20, 822; 2.964S, 78.70199W, 3140 m, QCAZ 825; Patacocha hill, 3.121109S, 79.065W, 3340 m, QCAZ 6144; Pucara, 3.21367S, 79.46739W, QCAZ 11038; Quinoas river, 3.087267S, 79.27762W, 3200 m, QCAZ 1564, 1566; Sigsig, 2.99900S, 78.80700W, 2890 m, QCAZ 1537; 3.129500S, 78.80400W, 2969 m, QCAZ 5605–5606, 5608; Sigsig-Gualaquiza, 3.106875S, 78.79558W, 2935 m, QCAZ 8646–8647; Tarqui, 3.015880S, 79.04447W, 2680 m, QCAZ 8512. **Provincia Cañar**: Cajas National Park, 2.70654S, 79.22765W, 3651 m, QCAZ 8946; Cañar, 2.560760S, 78.93077W, QCAZ 9947; Guallicanga river, 2.473189S, 78.97289W, 3048 m, QCAZ 10051–53; Gualaceo, 2.882159S, 78.77536W, 2298 m, QCAZ 9606; Juncal, 2.432109S, 78.90223W, 3960 m, QCAZ 10048; 2.473189S, 78.97289W, 3048 m, QCAZ 10050; Mazar, 2.54508S, 78.70078W, 2839 m, QCAZ 15811–13; 2.54649S, 78.69826W, 2924 m, QCAZ 15814–16; 2.57138S, 78.746W, 3442 m, QCAZ 15817–15823; 2.5708S, 78.74586W, 3451 m, QCAZ 15824; 2.545804S, 78.69611W, 2842 m, QCAZ 10970. **Provincia Chimborazo**: Frutatián lake, 2.21584S, 78.50136W, 3700 m, QCAZ 9217–9218; Magdalena lake, 2.187416S, 78.50686W, 3556 m, QCAZ 9214; Ozogoche, 2.368733S, 78.68871W, 4040 m, QCAZ 6006; Riobamba-Melán, 1.875020S, 78.54773W, 3564 m, QCAZ 9626–9628; Riobamba-Timbo, 1.929219S, 78.53718W, 3408 m, QCAZ 9616–9620; Shulata, 2.339309S, 78.84322W, 3228 m, QCAZ 5597–5598. **Provincia El Oro**: Guanazán, 3.440034S, 79.48695W, 2638 m, QCAZ 7894. **Provincia Loja**: Fierro Hurco, 3.710421S, 79.30498W, 3439 m, QCAZ 6949–6950; Jimbura-Jimbura lake, 4.708868S, 79.44657W, 3036 m, QCAZ 6947–6948; Jimbura- path to Jimbura lake, 4.709469S, 79.43558W, 3348 m, QCAZ 10054–10055, 10057–10062; Jimbura-Lagunillos, 4.628244S, 79.46353W, 3450 m, QCAZ 6146–6147; 4.817000S, 79.36199W, 3600 m, QCAZ 3785; San Lucas, 3.731853S, 79.26059W, 2470 m, QCAZ 2861; Saraguro, 3.62025S, 79.23581W, 3100 m, QCAZ 3606; 3.679457S, 79.23769W, 3190 m, QCAZ 3673–3674; Tarqui, QCAZ 5545. **Provincia Morona Santiago**: Sangay National Park, 1.960939S, 78.43198W, 3345 m, QCAZ 9612. **Provincia Tungurahua**: Patate-El Corral, 1.2725S, 78.46805W, 3468 m, QCAZ 9995–9996. **Provincia Zamora Chinchipe**: Podocarpus National Park, 4.484149S, 79.14875W, 1800 m, QCAZ 3743.
